# Local Inflammation Induces Complement Crosstalk Which Amplifies the Antimicrobial Response

**DOI:** 10.1371/journal.ppat.1000282

**Published:** 2009-01-30

**Authors:** Jing Zhang, Jingyun Koh, Jinhua Lu, Steffen Thiel, Benjamin S. H. Leong, Sunil Sethi, Cynthia Y. X. He, Bow Ho, Jeak L. Ding

**Affiliations:** 1 NUS Graduate School for Integrative Science and Engineering, National University of Singapore, Singapore; 2 Department of Biological Sciences, Faculty of Science, National University of Singapore, Singapore; 3 Department of Microbiology, Yong Loo Lin School of Medicine, National University of Singapore, Singapore; 4 Department of Medical Microbiology and Immunology, University of Aarhus, Aarhus, Denmark; 5 Emergency Medicine Department, National University Hospital, Singapore; 6 Department of Pathology, Yong Loo Lin School of Medicine, National University of Singapore, Singapore; University of Toronto, Canada

## Abstract

By eliciting inflammatory responses, the human immunosurveillance system notably combats invading pathogens, during which acute phase proteins (CRP and cytokines) are elevated markedly. However, the *Pseudomonas aeruginosa* is a persistent opportunistic pathogen prevalent at the site of local inflammation, and its acquisition of multiple antibiotic-resistance factors poses grave challenges to patient healthcare management. Using blood samples from infected patients, we demonstrate that *P. aeruginosa* is effectively killed in the plasma under defined local infection-inflammation condition, where slight acidosis and reduced calcium levels (pH 6.5, 2 mM calcium) typically prevail. We showed that this powerful antimicrobial activity is provoked by crosstalk between two plasma proteins; CRP∶L-ficolin interaction led to communication between the complement classical and lectin pathways from which two amplification events emerged. Assays for C4 deposition, phagocytosis, and protein competition consistently proved the functional significance of the amplification pathways in boosting complement-mediated antimicrobial activity. The infection-inflammation condition induced a 100-fold increase in CRP∶L-ficolin interaction in a pH- and calcium-sensitive manner. We conclude that the infection-induced local inflammatory conditions trigger a strong interaction between CRP∶L-ficolin, eliciting complement-amplification pathways which are autonomous and which co-exist with and reinforce the classical and lectin pathways. Our findings provide new insights into the host immune response to *P. aeruginosa* infection under pathological conditions and the potential development of new therapeutic strategies against bacterial infection.

## Author Summary

An opportunistic pathogen, *Pseudomonas aeruginosa* causes local inflammation in various tissues and is an important cause of hospital-acquired infections. While this may manifest as a hot-tub rash, failure to limit such localized inflammation may cause life-threatening septic shock to ensue, leading to multiple organ failure and possibly death. Effective treatment with antibiotics is complicated by the increasing prevalence of multidrug-resistant *P. aeruginosa*. In this study, we demonstrated that under typical local infection-inflammation conditions of slight acidosis and reduced calcium levels in the blood, *P. aeruginosa* is killed effectively. This is because such a milieu provokes crosstalk between two proteins involved in host defense, CRP and L-ficolin, which triggers communication between antimicrobial complement pathways that were previously presumed independent of each other. The CRP∶L-ficolin interaction amplifies their antimicrobial activity and enhances bacterial clearance. Insights gained from this host–pathogen interaction mechanism offer fresh perspectives on the potential development of complement immune therapies to enhance eradication of the invading pathogen.

## Introduction

The human immune system is notable for its ability to combat infectious microorganism by eliciting inflammatory responses [1]. Acute phase proteins such as CRP [2],[3] and cytokines [4] are elevated markedly in association with infection and inflammation. During this process, local acidosis occurs due to massive infiltration of neutrophils and macrophages [5] to the site of infection, which subsequently, activates the respiratory burst [6],[7] in many infection-inflammation related diseases such as trauma-induced infection [8], acute renal failure [9], and intra-abdominal infection [10]. These pathological conditions can decrease the pH to as low as 5.5–7.0 [11]. Simultaneously, mild hypocalcaemia is closely associated with bacterial infection [12]. One possibility is that intracellular NF-κB activation requires the influx of calcium into the immune cells [13], which is strengthened under acidic condition [14],[15], causing a transitory drop in the extracellular calcium at the infection site. The transient changes in the (i) levels of acute phase proteins, (ii) local pH and (iii) calcium concentration, all contribute to a typical infection-inflammation environment generally proposed to result from pathogenic metabolic disorder [16]. However, the concurrence of the infection-inflammation and the changes in pH, calcium and acute phase protein concentrations indicate that these pathophysiological conditions might also be essential for effective host defense.

Emerging evidence has demonstrated that a transient drop in pH and calcium level is crucial for triggering many immune processes, for example, TLRs 3, 7, 8 and 9 all require an acidic environment for their activation of the endosomes [17]. Extracellular acidosis was also found to boost adaptive immunity by activating dendritic cells [18], and CD8+ T cells in the peripheral tissues to improve MHC class I-restricted Ag presentation by neutrophils [19],[20]. However, complement activity, which is the major frontline host defense expected to occur under such typical infection-inflammation condition, is hardly explored even though it elicits a more rapid and direct antimicrobial action against the invading bacteria.

CRP and ficolins are known initiators of the complement classical pathway [3] and lectin pathway [21], respectively, and they are the key molecules that boost the immune response [22]. As an acute phase inflammation marker [23], CRP is also a multifunctional protein [3] upregulated in many diseases such as acute pneumonia [24], myocardial infarction [25] and atherothrombosis [26]. Besides binding to a wide range of ligands including phosphorylcholine (PC), polycations and polysaccharides displayed on the surface of the invading bacteria [27],[28], the CRP was also found to be deposited at the site of injury [2] indicating its crucial role in local inflammation. Similarly, as a pattern recognition receptor (PRR), ficolin which is composed of collagen-like domain and fibrinogen-like domain (FBG), recognizes lipoteichoic acid [29] of Gram positive bacteria; lipopolysaccharide of Gram negative bacteria; and 1,3-β-D-glucan of fungi; via the acetyl group on the N-acetylglucosamine (GlcNAc) moiety of these pathogen-associated molecular patterns [22],[30],[31]. There are three ficolin isoforms; L- and H- ficolins are soluble serum proteins whereas M-ficolin is mainly associated on the monocyte membrane, with very low concentration (∼60 ng/ml) in the serum [32]. Patients with ficolin disorder are susceptible to inflammation brought about by respiratory infections [33] and Behçet's disease [34]. Importantly, both CRP and ficolin undergo calcium- and pH- regulated conformational change when binding to their respective ligands [35],[36], indicating that their functions might be modulated by inflammation. However, no pathophysiological significance was proposed for CRP and ficolins although Ng et al. (2007) reported the functional relationship of their respective homologues, CrCRP and CL5, in the horseshoe crab.

As the most common cause of pneumonia in intensive care unit and the second most common cause of nosocomial pneumonia, the *Pseudomonas aeruginosa* is a ubiquitous opportunistic pathogen which easily overcomes immunocompromised patients. Its acquisition of multiple antibiobitic-resistance factors [37],[38] poses a grave challenge to drug manufacturers and patient healthcare management. Here, we found that *P. aeruginosa*, which thrives in local infections of the urinary tract [39], soft tissue [40], bone and joint [41], was effectively killed within 30 min in a typical infection-inflammation condition in contrast to normal condition. To determine the underlying mechanisms of the antimicrobial action, the interaction between ficolins and CRP was extensively characterized. Importantly, we demonstrated that L-ficolin and CRP collaborate through protein-protein crosstalk resulting in two amplification pathways which reinforce the classical and lectin complement pathways, and ultimately control downstream complement events like C4 deposition and opsonization of the microbe. These results emphasize the critical role of the typical “infection-inflammation condition” in provoking the complement amplification pathways, which the host uses to effectively fight against invading pathogens such as *P. aeruginosa*.

## Results

### Definition of the normal and infection-inflammation conditions

Calcium concentrations of 2 and 2.5 mM were employed to represent mild hypocalcaemia and normal conditions, respectively, since calcium concentration in healthy serum ranges from 2.2 to 2.6 mM [12] as compared to <2.12 mM [42] in the serum of patients with infection. pH 6.5 was reportedly typical of local acidosis [11]. CRP levels in normal (n = 5) and patient sera (n = 9) were measured and patients with acute phase infection recruited in this study typically showed serum CRP level of 10 µg/ml (Figure S1), which was used in subsequent experiments. Hence, two typical conditions were defined: (i) the infection-inflammation induced local acidosis (pH 6.5, 2 mM calcium in infected serum with CRP of 10 µg/ml) and (ii) normal condition (pH 7.4, 2.5 mM calcium in normal serum with CRP<0.5 µg/ml). Henceforth, these two conditions are referred as “normal” and “infection-inflammation” condition (mimicking local acidosis). Unless otherwise stated, the two buffers used to dilute the serum to mimic the normal and infection-inflammation conditions are: TBS buffer containing 25 mM Tris, 145 mM NaCl, pH 7.4 and 2.5 mM CaCl_2_ and MBS buffer containing 25 mM MES, 145 mM NaCl, pH 6.5 and 2 mM CaCl_2_ [43],[44].

### Infection-inflammation condition enhances bacterial killing by the synergistic action of CRP and L-ficolin

**Figure 1:**
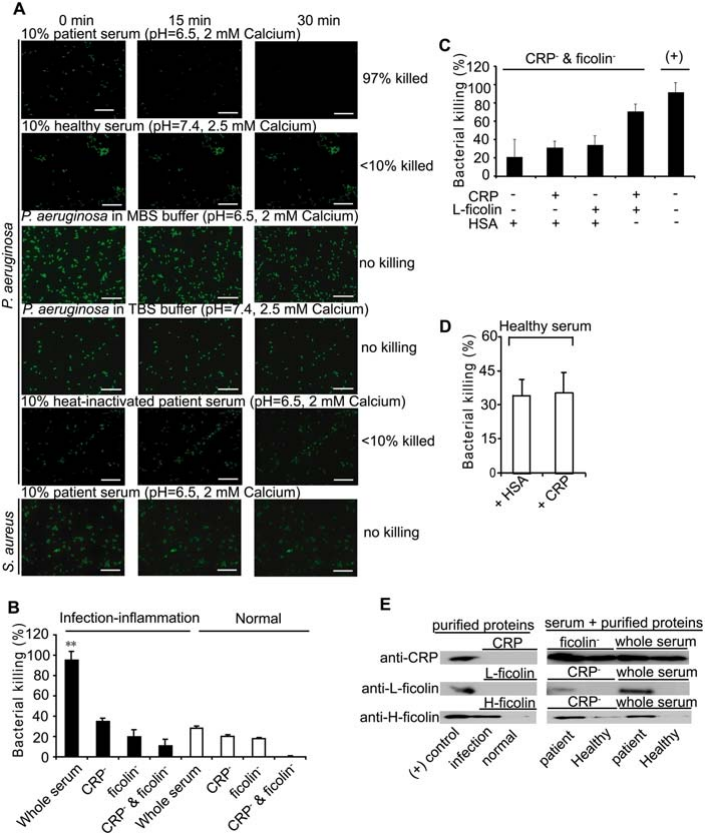
Infection-inflammation condition enhanced the bacterial killing effect of complement system by the synergistic action of CRP and L-ficolin. (A) Real-time observation of the bacterial killing effect of serum under normal (pH 7.4, 2.5 mM calcium) or infection-inflammation condition (pH 6.5, 2 mM calcium). The bactericidal effect of patient serum, healthy serum, and heat-inactivated patient serum on *P. aeruginosa* was tested and compared with the effect of TBS and MBS buffers. To show the specificity of the complement-mediated killing, the *S. aureus*, known to evade the complement system, was also tested. Images were taken at intervals of 15 s for 30 min at magnification of 63×1.6. The scale bar indicates 20 µm. (B) The end-point of the bacterial killing effect caused by the synergistic action of CRP and L-ficolin in whole serum, CRP-depleted serum (CRP^−^), ficolin-depleted serum (ficolin^−^), both CRP- and ficolin-depleted serum (CRP^−^ and ficolin^−^) under normal and infection-inflammation conditions, respectively. (C) The restoration of the bacterial killing effect by addition of CRP (10 µg/ml) or L-ficolin (3 µg/ml) or both of the proteins to both CRP- and ficolin-depleted serum (CRP^−^ & ficolin^−^) under normal and infection-inflammation conditions. To keep the total amount of protein constant, the reaction mixture was supplemented with HSA to top up the protein to 13 µg/ml. Bacteria incubated with undepleted serum (indicated as “(+)”) were used as positive control. (D) The potential bacterial killing effect by high CRP level was tested using healthy serum (at physiological pH and calcium level) supplemented with 10 µg/ml CRP. Bacteria incubated with healthy serum supplemented with same amount of HSA instead of CRP were the negative control. From (B) to (D), to exclude the influence of serum factors other than those of the complement system, bacteria incubated in heat-inactivated serum served as 100% viability count. Bacteria killed in the samples (compared to the heat-inactivated serum) were standardized as a percentage of the corresponding number of 100% live bacteria. (E) Binding of CRP and L-/H- ficolins to *P. aeruginosa*. Purified proteins including CRP and L-/H- ficolins together with patient or healthy serum, depleted or not depleted of ficolins or CRP, in TBS or MBS buffer were incubated with *P. aeruginosa* respectively. The bound proteins were eluted with 0.5 M ammonium formate and assayed for CRP, L-ficolin and H-ficolin by Western blot. The positive (+) control contained purified CRP or L- or H- ficolins loaded for confirmation of the specificity of immunodetection. **: *P*<0.01 by student's *t* test.

As a proof-of-concept and to demonstrate the prowess of the two typical conditions ascribed above, we compared the bactericidal activity against *P. aeruginosa*, a clinically challenging pathogen commonly found at the site of local inflammation. Figure 1A and Figure S2A show that within 30 min, under the infection-inflammation condition, 97% of the bacteria was killed in the patient serum (Video S1) whereas under the normal condition, <10% succumbed in the healthy serum (Video S2) indicating that the infection-inflammation condition elicits a highly robust antibacterial action. *P. aeruginosa* incubated with two buffers (TBS, pH 7.4, 2.5 mM CaCl_2_ or MBS, pH 6.5, 2 mM CaCl_2_) without serum remained viable (Video S3 and Video S4), suggesting that the enhanced bactericidal activity shown in Figure 1A was attributed to the serum components. Heat-inactivated patient serum restored the bacterial survival to 90% (Video S5), suggesting the involvement of the complement system, which is highly heat-sensitive. However, the patient serum failed to kill *Staphylococcus aureus* (Video S6), known to astutely evade the complement system [45], indicating that the local acidosis-mediated killing effect targets complement-susceptible pathogens.

To delineate the mechanisms of complement enhancement by infection-inflammation, we focused on the CRP and ficolins, two pH- and calcium- sensitive components of the complement system. Here, the antibacterial activity resulting from bacteria incubated with heat-inactivated serum was used as a background control to normalize off any potential effect of other heat-resistant serum factors. Figure 1B shows that within 1 h, the patient serum elicited >95% antibacterial activity compared to 30% by healthy serum whereas the two buffers (TBS or MBS) did not kill the bacteria (Figure S2B). However, depletion of either CRP or ficolin from the sera drastically decreased the antimicrobial activity to 20–30% for both of the healthy and patient sera, indicating that in the infection-inflammation condition, CRP and ficolin are necessary to trigger efficient bactericidal activity. Interestingly, we observed synergistic action of CRP and L-ficolin when both proteins were present in the patient serum under infection-inflammation condition, which accounted for ∼50% of the enhanced killing effect. To verify this synergistic effect, serum depleted of both CRP and ficolin (by PC- or GlcNAc- beads) was used for incubation with the bacteria during which increasing concentrations of CRP or L-ficolin or both of the proteins were added. The results confirmed that addition of the two proteins exhibited a significant amplification of bacterial killing compared to adding either of the two proteins, although this process did not restore the antimicrobial effect to the same level as that of the original undepleted serum (Figure 1C). This implies that some other serum factors might have been lost through their association with the GlcNAc or PC beads together with L-ficolin or CRP, and that they (for example mannose binding lectin [46]) might also contribute to the antimicrobial activity via complement pathways. To further confirm the significance of the infection-inflammation condition, normal healthy serum was supplemented with 10 µg CRP and incubated with *P. aeruginosa* to simulate a condition where higher CRP level prevails (but without infection) such as in cardiovascular disease. Our results indicated that without infection-inflammation condition, a high CRP level did not bring about antimicrobial activity (Figure 1D). Therefore, we envisaged potential interaction and/or co-operation between CRP and L-ficolin which might be strengthened under infection-inflammation condition, causing amplification of the complement activity and higher bactericidal activity. To ascertain this possibility, *P. aeruginosa* was incubated with either of the purified CRP and L-/H- ficolins (major serum-type ficolins) or with these purified protein plus serum components. Results showed that although neither of the purified CRP nor L-ficolin was bound directly to *P. aeruginosa* (in the absence of serum), the CRP was bound indirectly but equally well to *P. aeruginosa* in the patient or healthy serum independent of depletion of ficolins (Figure 1E). Furthermore, in patient serum under infection-inflammation condition, CRP enabled the indirect association of L-ficolin to the invading bacteria. This was confirmed by a drop in the level of bound L-ficolin in CRP-depleted serum. However, the purified H-ficolin on its own (used here for comparison with L-ficolin), was bound directly to the bacteria under infection-inflammation condition, independent of CRP (Figure 1E). The low homology (42%) between H-ficolin and L- ficolin (Figure S3), may explain the lack of interaction between H-ficolin and CRP. Thus, our data suggest: (i) the possibility of the PRR∶PRR interactions between L-ficolin and CRP, (ii) that in the healthy serum, only CRP binds to bacteria resulting in minimal complement activity, (iii) in infection-inflammation condition, CRP enables L-ficolin to bind bacteria, which upregulates the complement pathway.

### Infection-inflammation triggers CRP and L-ficolin to form a complex in the serum

**Figure 2:**
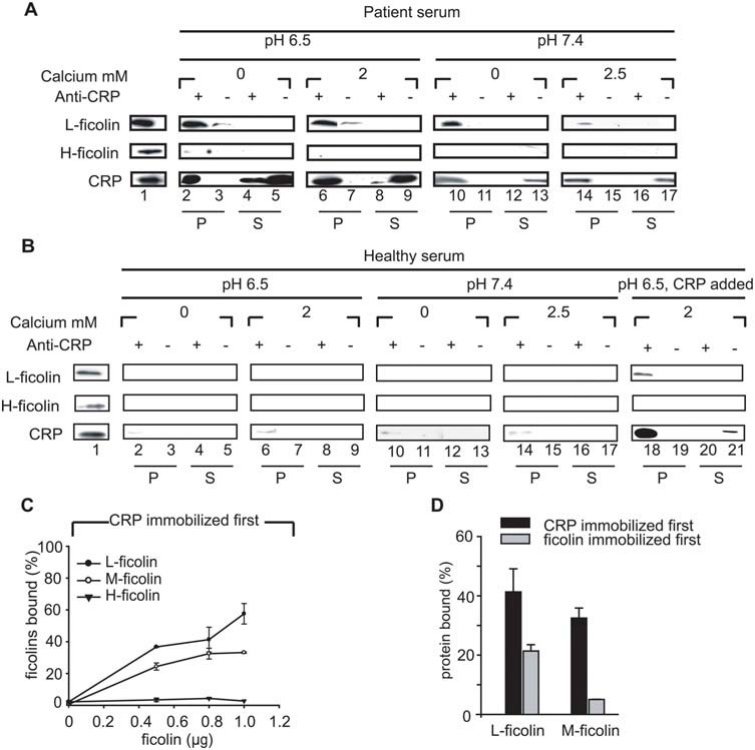
Infection-inflammation triggers CRP and L-ficolin to form complex in serum. The complex of CRP and L-ficolin was co-immunoprecipitated from the patient serum (A) or healthy serum (B) at pH 6.5 and 7.4 with or without calcium. Rabbit polyclonal anti-CRP was added (lanes marked with “+”) to 500 µl of 10% pre-cleared serum and incubated for 1 h at 4°C, following which protein A beads were added. As the negative controls (lanes marked with“−”), the Co-IP used rabbit polyclonal anti-CrCRP (horseshoe crab, *Carcinoscorpius rotundicauda* CRP antibody) instead of rabbit polyclonal anti-CRP. The immuno-precipitates (P) on the beads and the proteins in the supernatants (S) were extracted in SDS PAGE loading buffer, and the bound CRP and L-/H-ficolins were identified by mouse monoclonal anti-CRP and anti-L-/H-ficolin antibodies, respectively. In (A) and (B), Lane 1: purified CRP and L-/H-ficolins were loaded for confirmation of the specificity of immunodetection; Lanes 2–5: proteins in precipitates or supernatants under pH 6.5 without calcium; Lanes 6–9: proteins in precipitates or supernatants under pH 6.5 with 2 mM calcium. Lanes 10–13: proteins in precipitates or supernatants under pH 7.4 without calcium; Lanes 14–17: proteins in precipitates or supernatants under pH 7.4 with 2.5 mM calcium. In (B), Lanes 18–21 contained proteins in precipitates or supernatants under pH 7.4, 2.5 mM calcium with addition of 10 µg/ml CRP. (C) ELISA to test the interaction between CRP and L-/H-/M-ficolins when 0.8 µg of CRP was immobilized first. 0, 0.5, 0.8 and 1 µg of L-/H-/M-ficolins were added to the immobilized CRP, respectively, and the bound proteins were detected by the corresponding antibodies. (D) ELISA to compare the interaction between CRP and L-/M-ficolins in different orders of immobilization with 0.8 µg of either of the ficolins or CRP immobilized first. In (C) and (D), 0.8 µg HSA (instead of CRP or ficolins) was immobilized on the wells to serve as the negative control. The positive control (for 100% binding) constituted 1 µg of the binding proteins [ficolins in (C) and ficolins or CRP in (D)] directly immobilized on the wells and detected by the corresponding antibodies. OD_405 nm_ was read as the binding signal. Readings were subtracted off negative controls and expressed as a percentage of the corresponding 100% binding control.

Based on the *in vitro* bactericidal results we hypothesized that CRP and L-ficolin might interact in the patient's serum under infection-inflammation condition with mild acidosis. Thus, it was imperative for us to investigate whether CRP has a propensity to complex with L-ficolin in the patient serum. To delineate the effect of different serum factors, we tested the potential interaction between CRP and L-ficolin by varying the pH, calcium and CRP levels. Consistent with the antibacterial results, co-immunoprecipitation (Co-IP) of the patient sera under pH 6.5 showed strongest complexes of CRP∶L-ficolin (Figure 2A) independent of the calcium level. However, at pH 7.4, increasing calcium was found to dramatically inhibit the CRP∶L-ficolin interaction, indicating that under normal condition, calcium prevents CRP∶L-ficolin interaction. Furthermore, the protein complex could not be isolated from the healthy serum regardless of pH and calcium status (Figure 2B, lanes 2–17). This might be because healthy individuals have a dramatically lower CRP level (100–1000× less than acute phase patients; see Figure S1). To confirm this, we reconstituted the healthy serum with 10 µg/ml CRP (acute phase level) and adjusted the pH and calcium to infection-inflammation condition. Indeed, we were able to isolate CRP∶L-ficolin complex from the simulated patient serum (Figure 2B, lanes 18–21). In contrast, H-ficolin was shown not to complex with CRP *in vivo*, regardless of conditions (Figure 2A and 2B), indicating the specificity of CRP for its cognate ficolin partners.

To substantiate that CRP and ficolins interact directly, the three ficolin isoforms, which share some common functions such as complement activation [47]–[49], were purified (Figure S4) and ELISA was used to test their interaction status with CRP. Figure 2C shows that only L- and M- ficolins exhibited dose-dependent binding to immobilized CRP whereas H-ficolin, which is independent of calcium and orders of immobilization, did not associate with CRP (Figure S5A). On the premise that both ficolins and CRP can anchor to the invading bacteria directly or indirectly [22],[29], we also analyzed the reverse orders of binding by immobilizing the ficolins first to a solid phase followed by addition of CRP. Figure 2D shows that the interactions between CRP and L-/M-ficolins were weaker when ficolins were immobilized first indicating that prior immobilization of CRP appeared to be a preferred position/orientation for subsequent binding of ficolins. This is consistent with our observation that after anchorage, CRP might recruit ficolin to the bacterial surface (Figure 1D). Our observation that M-ficolin also bound to immobilised CRP (Figure 2C), prompted us to perform yeast two-hybrid analysis (Figure S5B) to confirm their interaction since M-ficolin, the major membrane-associated form of ficolin, has also been implicated in complement activation [47] and phagocytosis of pathogens [50]. Our results show that besides serum L-ficolin∶CRP interaction, the M-ficolin∶CRP interaction also occurred (Figure S5B), and that this partnership is probably significant to intracellular/downstream activities.

### CRP∶L-ficolin interaction triggers two new complement amplification pathways which upregulate opsonization and phagocytosis

**Figure 3:**
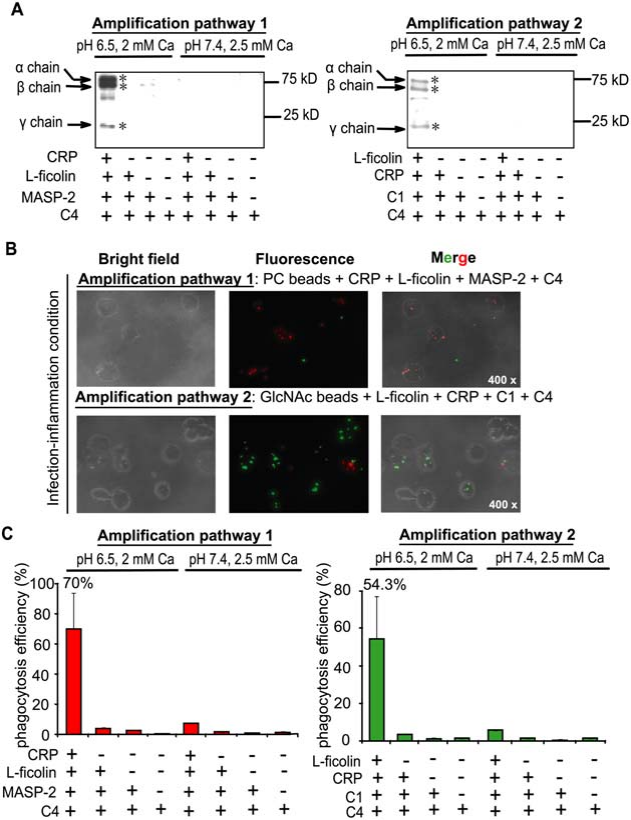
Downstream functional analysis of amplification pathways 1 and 2 by C4 cleavage assay and phagocytosis assay. (A) C4 cleavage assay with PC and GlcNAc as ligands in normal (pH 7.4, 2.5 mM calcium) and infection-inflammation (pH 6.5, 2 mM calcium) conditions. PC beads were incubated with amplification pathway 1 components (CRP, L-ficolin, MASP-2, or C4) and GlcNAc beads were incubated with amplification pathway 2 components (L-ficolin, CRP, C1, or C4) under normal and infection-inflammation conditions. The cleaved C4 deposited on the beads were detected by Western blot. Negative controls were beads incubated with complete amplification components under normal condition, and incomplete amplification pathway components under normal or infection-inflammation conditions. * indicates the C4 cleavage products. (B) The phagocytosis assay of the opsonized beads generated in amplification pathways 1 and 2 under infection-inflammation condition (magnification: 400×). The fluorospheres with PC-BSA (red) or GlcNAc-BSA (green) immobilized on the surface were opsonized by incubating with complete or partial components of the amplification pathway as indicated in the C4 cleavage assay (under infection-inflammation condition). These beads (red or green) were mixed with equal amounts of control beads (green or red) which have BSA immobilized on their surfaces without PC- or GlcNAc-residues. The mixture (opsonized beads∶control beads∶U937 cells) at a ratio of 15∶15∶1 was incubated at 37°C for 30 min with gentle shaking. Cells fixed in 4% paraformaldehyde were visualized under fluorescence microscope. (C) Quantification of the phagocytic efficiency observed under the microscope. Three different fields were chosen for each sample and the average net number of opsonized beads (number of opsonized beads−number of control beads) in each cell in each field was enumerated and calculated. A count of 15 beads in each cell was considered 100% phagocytic efficiency.

As both CRP and ficolins are hitherto well known to separately trigger the classical and lectin complement pathways, we hypothesize that the interaction between CRP and L-ficolin under infection-inflammation condition might connect these two pathways and potentially ramify new conduits to potentiate the bactericidal activity in patients with infection-induced mild local acidosis. Thus, C4 cleavage assay was performed to investigate the complement activity via sequential incubation of PC-beads with CRP, L-ficolin, MASP-2 and C4 (Figure 3A, left panel) or GlcNAc-beads with L-ficolin, CRP, C1 complex and C4 (Figure 3A, right panel). The C4 cleavage product, C4b (α,β,γ, chains), resulting from both of the reactions only emerged under infection-inflammation condition. These results suggest that both orders of interactions: L-ficolin∶CRP and CRP∶L-ficolin produced functional complement components under pathophysiological conditions although prior anchorage of CRP was apparently the preferred position for interaction with L-ficolin (Figure 1E and Figure 2D). To confirm that the opsonized particle generated by interaction between CRP and L-ficolin can be phagocytosed, and to avoid background interaction between other receptors directly/independently with pathogen-associated molecular patterns on the bacterial membrane, we incubated the above C4-deposited beads rather than opsonized bacteria with phagocytes. Consistent with the C4 deposition results, only beads with all the components of the amplification pathway added in the infection-inflammation condition underwent significant opsonization and phagocytosis within 15 min (Figure 3B and Figure S6). Under infection-inflammation condition, the amplification pathways 1 and 2 induced phagocytic efficiencies up to 70% and 54.3%, respectively, with <5% in the other controls (Figure 3C). Taken together, we propose that inflammation drives crosstalk between CRP and L-ficolin from which two new autonomous complement amplification pathways emerge leading to membrane attack complex (MAC) formation and antimicrobial activity:

- Amplification pathway 1: Infection by Gram positive bacteria containing PC (a chemical moiety of lipoteichoic acid) induces the CRP∶L-ficolin mediated regulation of the classical pathway: *PC→CRP∶L-Ficolin→MASP-2→C4→C3→MAC*
- Amplification pathway 2: Infection by Gram negative bacteria containing GlcNAc (a chemical moiety of lipopolysaccharide) induces the L-ficolin∶CRP mediated regulation of the lectin pathway: *GlcNAc→L-Ficolin∶CRP→C1q→C4→C2→C3→MAC*

### L-ficolin∶CRP and CRP∶L-ficolin interactions integrate the classical and lectin complement pathways to enhance antimicrobial activity

**Figure 4:**
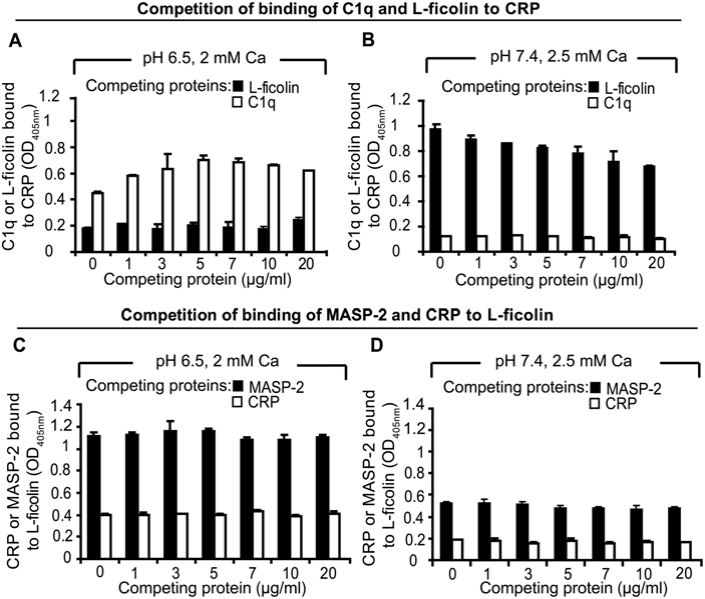
Co-existence of the amplification pathways and the classical and lectin pathways. (A) Competition of binding of C1q and L-ficolin to CRP at pH 6.5 and 2 mM calcium. 0.8 µg of C1q mixed with increasing amounts of L-ficolin (black bars) or 0.8 µg of L-ficolin mixed with increasing amount of C1q (white bars) in blocking buffer (MBS with 0.05% Tween-20 and 1% HSA, pH 6.5) was added to the immobilized CRP. The amount of bound C1q (black bars) or L-ficolin (white bars) was detected by the corresponding antibodies. (B) Competition of binding of C1q and L-ficolin to CRP at pH 7.4 and 2.5 mM calcium. 0.8 µg of C1q mixed with increasing amount of L-ficolin (black bars) or 0.8 µg of L-ficolin mixed with increasing amount of C1q (white bars) in blocking buffer (TBS with 0.05% Tween-20 and 1% HSA, pH 7.4) was added to the immobilized CRP. The bound C1q (black bars) or L-ficolin (white bars) was detected by the corresponding antibodies. (C) Competition of binding of MASP-2 and CRP to L-ficolin at pH 6.5 and 2 mM calcium. 0.8 µg of CRP mixed with increasing amount of MASP-2 (black bars) or 0.8 µg of MASP-2 mixed with increasing amount of CRP (white bars) in blocking buffer (MBS with 0.05% Tween-20 and 1% HSA, pH 6.5) was added to the immobilized L-ficolin. The bound CRP (black bars) or MASP-2 (white bars) was detected by the corresponding antibodies. (D) Competition of binding of MASP-2 and CRP to L-ficolin at pH 7.4 and 2.5 mM calcium. 0.8 µg of CRP mixed with increasing amount of MASP-2 (black bars) or 0.8 µg of MASP-2 mixed with increasing amount of CRP (white bars) in blocking buffer (TBS with 0.05% Tween-20 and 1% HSA, pH 7.4) was added to the immobilized L-ficolin. The bound CRP (black bars) or MASP-2 (white bars) was detected by the corresponding antibodies. 0.8 µg of HSA immobilized on the wells instead of CRP was the negative control and readings were subtracted off the negative controls. Ca annotates calcium.

To determine whether C1q and L-ficolin can bind to CRP simultaneously and which of the two amplification pathways is dominant, competition assay was performed. Under either infection-inflammation or normal condition, the addition of increasing amounts of L-ficolin to a fixed concentration of C1q did not dissociate C1q from CRP and vice versa (Figure 4A and 4B) indicating that C1q and L-ficolin might bind to different domains of the CRP molecule. Similarly, under either normal or infection-inflammation condition, CRP and MASP-2 did not compete with each other for L-ficolin (Figure 4C and 4D). Interestingly, under infection-inflammation condition, we detected a 5-fold increase in the CRP∶L-ficolin interaction but the CRP∶C1q interaction was reduced to half compared to physiological condition. In comparison, both the binding between L-ficolin∶MASP-2 and L-ficolin∶CRP became significantly increased in infection-inflammation condition indicating that the activities of the four pathways (classical, lectin, amplication 1 and amplification 2) might be tightly regulated to kill the pathogens effectively. Overall, the two amplification pathways do not compete against the classical and lectin-mediated pathways, rather, they integrate and boost the classical and lectin-mediated complement pathways towards a common goal of overcoming the pathogen more effectively while avoiding complement over-reaction.

### Biochemical characterization of the interaction between CRP and L-ficolin

**Figure 5:**
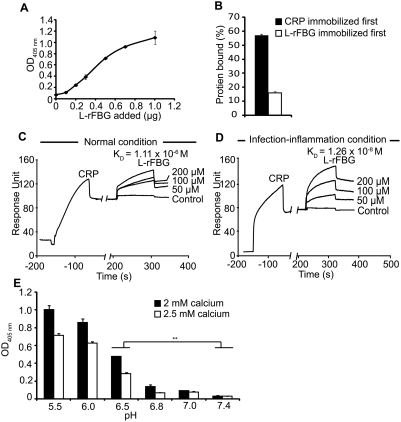
Biochemical characterization of the interaction between CRP and L-rFBG. (A) Dose-dependent binding of L-rFBG to 0.8 µg of immobilized CRP. The indicated amount of L-rFBG in blocking buffer was added to the immobilized CRP. OD_405 nm_ was read to indicate the binding of L-rFBG to CRP. 0.8 µg of HSA immobilized on the wells instead of CRP was the negative control and readings were subtracted off the negative controls. (B) Effect of the order of immobilization on the binding between CRP and L-rFBG. 0.8 µg of L-rFBG was added to 0.8 µg of immobilized CRP (black bar) and 0.8 µg of CRP was added to 0.8 µg of immobilized L-rFBG (white bar). To compare the data from the two orders of immobilization, the binding proteins (L-rFBG or CRP) directly immobilized on the plate were detected and were taken as 100% binding, to which other readings were normalized against. The affinity between CRP and L-rFBG under normal (C) or infection-inflammation condition (D) was tested by surface plasmon resonance analysis using BIAcore 2000. POPC was immobilized on the HPA chip to expose PC as ligand for CRP. A 50 µl of CRP at 200 nM in the running buffer was injected. Fifty µl aliquots of L-rFBG at the indicated concentrations were injected afterwards. The negative controls involved injection of buffers (without L-rFBG) over the PC-HPA chip immobilized with CRP. Data were analyzed using BIAevaluation 3.2 software. (E) Binding of L-rFBG to immobilized CRP at pH ranging from 5.5 to 7.4, in the presence of 2 mM calcium (black bars) or 2.5 mM calcium (white bars). HSA instead of CRP immobilized on the plates was the negative control and readings were subtracted off the negative controls.

To delineate the mechanism underlying the CRP∶L-ficolin interaction induced by the infection-inflammation condition, the molecular interaction between these two proteins were analyzed. Given that ficolins are composed of FBG and collagen-like domains, and that the FBG domain harbors the ligand binding sites [51],[52], we hypothesize that CRP binds to the FBG domain of L-ficolin. To prove this, the recombinant FBG domain of L-ficolin (henceforth referred as L-rFBG) was expressed and purified (Figure S4). ELISA showed that L-rFBG displayed dose-dependent binding to CRP immobilized on Maxisorp™ plates (Figure 5A), indicating that CRP binds to the L-rFBG. However, when L- rFBG was immobilized first, its binding of CRP was weaker (Figure 5B), which is consistent with our observation that prior anchorage of CRP to the bacterial surface is the preferred position for the CRP∶ficolin interaction (Figure 2D). Real-time biointeraction analysis showed binding affinity between CRP∶L-rFBG to be 1.11×10^−6^ M under normal condition whereas infection-inflammation condition induced a 100-fold increase in their affinity (K_D_ = 1.26×10^−8^ M) (Figure 5C and 5D), which triggers two amplification pathways. The lower binding affinity between CRP and L-ficolin under normal condition suggests that under physiological condition, the two proteins only co-exist and not interact so as to avoid random complement activation.

To demonstrate how infection-inflammation condition triggers the interaction between CRP and ficolins, we examined their binding characterisitics over a range of pH 5.5 to 7.4 under either 2 mM or 2.5 mM calcium. Considering that prior anchorage of CRP is the preferred position for the recruitment of L-rFBG (Figure 5B), we immobilized CRP first. Figure 5E shows that the interaction between CRP and L-rFBG was influenced dramatically by pH, with stronger binding under acidic condition, implying that CRP∶L-ficolin interaction is triggered by the infection-inflammation condition whereas under normal condition, the CRP and L-ficolin co-exist with very weak or no complexation, possibly to avoid undesirable complement-enhancement. Furthermore, it was observed that interaction under 2 mM calcium showed stronger binding compared to that under 2.5 mM calcium. This is consistent with the previous observations that calcium influences the conformations of CRP and ficolins [51],[53] and suggests that blood calcium concentration may regulate the recruitment of ficolin to the CRP when the later is anchored on the bacterial surface. Overall, our data indicate that CRP∶L-ficolin interaction is enhanced by low pH and low calcium, which occur under infection-inflammation condition. Thus it is possible that CRP anchored to the invading bacteria is recognized by L-ficolin, and their interaction potentially activates the amplification pathways.

### Inflammation-induced CRP∶ficolin crosstalk increases C3 deposition to enhance killing of *P. aeruginosa*

**Figure 6:**
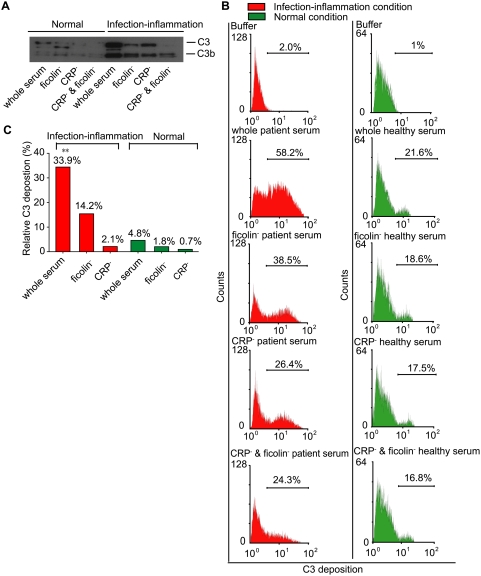
Crosstalk between CRP and ficolins generated more C3 deposition on the *P. aeruginosa* under the infection-inflammation condition. 10^8^ cfu bacteria were incubated with whole serum, CRP-depleted (CRP^−^), ficolins-depleted (ficolin^−^), or CRP- and ficolin-depleted sera (CRP^−^ & ficolin^−^) under normal or infection-inflammation conditions. The bound proteins were detected by Western blot (A) and flow cytometry (B). In (A), the bound proteins were eluted with 0.5 M ammonium formate and detected by Western blot. In (B), after incubation with serum, the bacteria were stained with primary antibody (anti-C3d at 1∶100) and secondary antibody (R-phycoerythrin-conjugated goat anti-rabbit at 1∶100). The signal was collected using Cytomation Cyan LX. The percentage of the cells that shifted is indicated in the curves. (C) Quantification of the opsonized bacteria collected by flow cytometry under different conditions. The background reading was taken from bacteria incubated with CRP- and ficolin-depleted serum. The percentage of C3-deposition was calculated by subtracting the background reading and plotted together. **: P<0.01 by student's t test.

At this juncture, it was imperative for us to show whether the two amplification pathways bridged by CRP∶ficolin crosstalk were responsible for the enhanced bactericidal activity of *P. aeruginosa* under infection-inflammation condition (see Figure 1). Since C3-deposition is the pivotal step towards the formation of the MAC, we sought to detect C3 deposition on the bacteria by incubating *P. aeruginosa* with whole serum, or serum depleted of CRP or ficolin in the healthy or infection-inflammation conditions. Figure 6A shows that bacteria incubated with patient serum in infection-inflammation condition had higher C3 deposition compared to normal serum. However, when the serum was depleted of CRP or ficolin, C3 deposition was reduced dramatically even in infection-inflammation condition, resulting in very little difference in the level of C3-deposition under the two conditions. This indicates that: (i) the presence of CRP and ficolin, and (ii) their interaction, were crucial to C3-deposition particularly in the infection-inflammation condition. Furthermore, after subtracting readings from the both the CRP- and ficolin- depleted serum (to exclude the effects of other serum factors), flow cytometry quantified a 7-fold greater C3-deposition on the *P. aeruginosa* induced by CRP∶L-ficolin interaction in the whole patient serum (Figure 6B) compared to that in the whole normal serum (Figure 6C). Furthermore, stepwise depletion of ficolin and CRP from the patient serum led to progressive loss in C3-deposition. Interestingly, in infection-inflammation condition, the combined presence of CRP and ficolin in the patient serum induced 58.2% C3-deposition compared to 24.3% with serum depleted of both CRP and ficolin (Figure 6B), a difference of 33.9% (Figure 6C). In contrast, CRP- or L-ficolin- depleted serum only induced 2.1% or 14.2% more deposition of C3, respectively (Figure 6C). Remarkably, the increase in C3-deposition in the presence of CRP and L-ficolin under infection-inflammation condition (pH 6.5, 2 mM calcium) was higher than the sum of the increased effect from either of the two individual proteins in the serum, demonstrating synergistic effect of the CRP∶ficolin interaction, and the enhancement of the complement activity against the invading bacteria. This is the underlying reason for the more efficient killing of the bacteria in such local inflammation condition. In healthy individuals, we envisage that these two molecules only co-exist and do not interact with each other, thus preventing random amplification of the complement activity, which would otherwise be detrimental to the host.

## Discussion

Local acidosis, mild hypocalcaemia and high CRP level are characteristic of local inflammation. By defining a typical infection-inflammation condition (pH 6.5 and 2 mM calcium), under which the serum kills the *P. aeruginosa* more effectively compared to normal physiological condition (pH 7.4 and 2.5 mM calcium), we demonstrated crosstalk between CRP and ficolin, which resulted in two new autonomous amplification pathways leading to a synergistic level of C3-deposition on the bacteria. This serves to boost the bactericidal activity in the serum under local infection-inflammation.

The complement system is a major microbicidal force in the infection-inflammation condition (Figure 1A) mediated by the synergistic effect of CRP and ficolins (Figure 1B–1D). We propose that CRP interacts with L-ficolin under infection-inflammation condition, enhancing the complement-activated killing of the *P. aeruginosa*. Although it was shown that purified CRP and L-ficolin did not bind to *P. aeruginosa* (Figure 1E), the presence of serum factors enabled CRP to bind to bacteria indirectly and the bound CRP recruited L-ficolin (Figure 1E). Co-IP confirmed that the CRP∶L-ficolin complex formation was induced in the serum by infection-inflammation condition (low pH, low calcium level and high CRP level) (Figure 2A and 2B) and ELISA demonstrated that L- and M- ficolins displayed stronger binding to CRP when the latter was anchored first to bacteria (Figure 2C and 2D).

Hitherto, two complement pathways involving CRP and L-ficolin are well-established [21],[28]. Here, we provide evidence for the inflammation-induced crosstalk between CRP and L-ficolin which impels two new amplification pathways:

1. *Amplification pathway 1* PC→CRP→L-ficolin→MASPs→C4→C3→MAC
2. *Amplification pathway 2* GlcNAc→L-ficolin→CRP→C1q,r,s→C4→C3→MAC

The cleavage of C4 (Figure 3A) demonstrated that these two amplification pathways were functional only under infection-inflammation condition but are negligible in healthy condition. The opsonized particles generated by these amplification pathways were recognized and removed by the immune cells indicating the autonomy and effectiveness of the two new pathways (Figure 3B) which were further confirmed by quantification of the phagocytic efficiency (Figure 3C). Competition assays showed that the amplification pathways do not compete against the classical and lectin pathways (Figure 4), but rather, they boost these two well-known complement pathways.

To unequivocally demonstrate that the CRP∶L-ficolin interaction is triggered by infection-inflammation condition, we showed that the L-rFBG interacts avidly with CRP (Figure 5A) at K_D_ of 1.26×10^−8^ M under infection-inflammation condition compared to 1.11×10^−6^ M under normal condition (Figure 5C and 5D). This represents a 100-fold increase in affinity between the two proteins. A positional preference was evident, where prior anchorage of CRP resulted in a more efficient recruitment of the L-ficolin (Figure 5B). Low pH dramatically enhanced CRP∶L-ficolin interaction (Figure 5E), showing that inflammation-induced acidosis promoted stronger crosstalk between CRP and L-ficolin. Furthermore, lower level of calcium enhanced the CRP∶L-ficolin interaction (Figure 5E), thus indicating that calcium is a main regulator of the molecular crosstalk between L-ficolin and CRP. Taken together, our findings explain why and how a typical infection-inflammation condition provokes crosstalk between CRP and the L-ficolin, which boosts the complement system. This key mechanism is further confirmed by the fact that in infection-inflammation condition, the CRP∶L-ficolin amplification pathway caused more C3-deposition (Figure 6).

**Figure 7:**
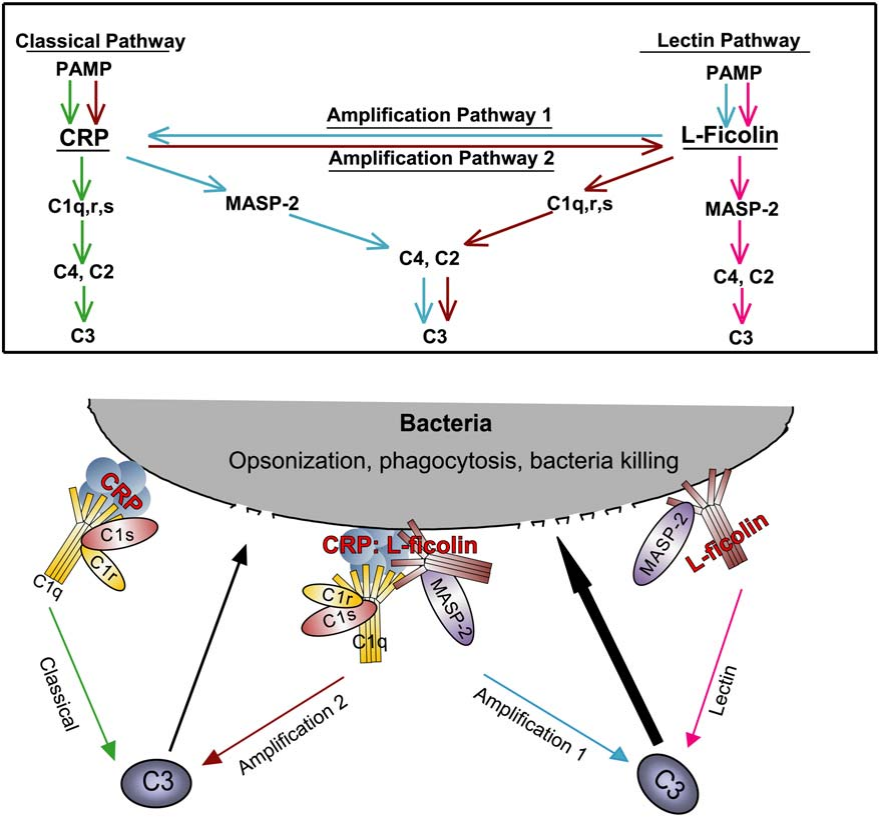
A model to illustrate the mechanism of enhanced antimicrobial activity of the serum in infection-inflammation condition. The upper panel illustrates the relationship between molecules involved in the classical pathway (green arrow), lectin pathway (red arrow), amplification pathway 1 (blue arrow), and 2 (brown arrow). In the lower panel, CRP and L-ficolin recruited to the bacterial surface by serum factors activate the classical and lectin complement pathways by interacting with C1 and MASP-2, which individually leads to the downstream events such as C4-cleavage and C3-deposition. The infection-inflammation condition triggers the CRP∶ficolin interaction, through which two autonomous amplification pathways emerge to boost antimicrobial activity of the classical (black upward arrow) and lectin-mediated (bold black upward arrow) complement pathways: (1) *Amplification pathway 1* (blue arrow): *PC→CRP→L-ficolin→MASPs→C4→C3→MAC*; (2) *Amplification pathway 2* (brown arrow): *GlcNAc→L-ficolin→CRP→C1q,r,s→C4→C3→MAC*.

In conclusion, we provide evidence that local infection-inflammation elicits new complement amplification mechanisms to fight the invading pathogen (Figure 7). This novel antimicrobial mechanism is particularly effective against the *P. aeruginosa*, an opportunistic pathogen which causes mortality in critically ill and immuno-compromised patients [39]. Both the immunoevasive nature [54] of *P. aeruginosa* as well as its acquisition of multi-drug resistance [55] makes elimination of this microorganism a particular challenge. To date, the effective therapy against *P. aeruginosa* infection remains indistinctive. However, insights gained into the mechanisms of complement amplification shown in the present work are crucial in understanding the host defense to counter pathogen immune evasion, which will contribute to the development of complement-immune therapies. Furthermore, the arena of complements is far from saturation and there are still intriguing forward and reverse defense mechanisms awaiting elucidation and understanding.

## Materials and Methods

### Ethics statement

All experiments were performed according to national and institutional guidelines on ethics and biosafety (Institutional Review Board, Reference Code: NUS-IRB 06-149).

### Proteins and sera

Human C1 complex, C4, CRP and polyclonal goat anti-human CRP antibody were purchased from Sigma. The purity of CRP was verified by SDS PAGE and Mass Spectrometry (Figure S7A and S7B). C1q was from Quidel (San Diego, CA). L- and H-ficolins were purified from human serum. Recombinant MASP-2 was expressed and purified according to Vorup-Jensen *et al.* [56]. M-ficolin and L-rFBG were recombinantly expressed and purified (in Protocol S1). Mouse anti-L- and H- ficolin monoclonal antibodies and rabbit anti-M-ficolin polyclonal antibody were from Hycult (Uden, The Netherlands). Goat anti-rabbit, rabbit anti-goat, goat anti-mouse secondary antibodies with HRP-conjugation, and polyclonal rabbit anti-C3d, anti-C4c and anti-C1q antibodies were from Dako A/S (Glostrup, Denmark). Goat anti-rabbit antibody with R-phycoerythrin conjugation and mouse anti-myc antibody were purchased from Invitrogen (Carlsbad, CA). Polyclonal goat anti-MASP-2 antibody was purchased from Santa Cruz Biotechnology (Santa Cruz, CA). Serum samples were obtained from adult male healthy and infected patient volunteers with informed consent. Clinical data indicated infection-inflammation in these patients. CRP- or ficolin- depleted serum was generated by incubating the serum with *p*-aminophenyl phosphorylcholine (PC)- or GlcNAc- Sepharose beads for 2 h at room temperature. The concentration of the sera was measured by NanoDrop™ ND-1000 Scientific, Wilmington spectrophotometer (Thermo Fisher Scientific, DE) to ensure that equal amounts of proteins were added. 10% (v/v) healthy serum was prepared by diluting the normal healthy serum in TBS buffer (25 mM Tris, 145 mM NaCl, pH 7.4, 2.5 mM CaCl_2_) buffer and 10% (v/v) patient serum was prepared by diluting the patient serum in MBS buffer (25 mM MES, 145 mM NaCl, pH 6.5, 2 mM CaCl_2_) unless otherwise stated. The CRP level was determined (Figure S1) using CRP Bioassay ELISA kit (BD Biosciences, San Jose, CA).

### Bacterial strains

*P. aeruginosa* strain PAO1 is commonly used in many studies including trials on multi-drug resistance [37],[38], simulation of *P. aeruginosa* infection [57],[58] and biofilm formation [59]. The lab-adapted PAO1 [60],[61] used in this study was kindly provided by Professor B. H. Iglewski (University of Rochester, Rochester, USA). The GFP functional fragment was cloned into the *Hind* III and *Sma* I sites in the pDSK 519 vector to form the pDSK-GFP plasmid which was subsequently transformed into PAO1 (PAO1-GFP). *S. aureus* strain PC1839 provided by Professor S. Arvidson (Karolinska Institutet, Stockholm, Sweden) was transformed with plasmid pALC 1420 (PC1839-GFP) [62]. The PAO1-GFP and PC1839-GFP which express enhanced GFP were used in the *in vitro* bacterial killing assay. The PAO1 without pDSK-GFP was used in flow cytometry experiment to avoid fluorescence quenching between different dyes.

### *In vitro* bacterial killing assay

Single colonies of *P. aeruginosa* PAO1 and *S. aureus* PC1839 were inoculated into 10 ml of Luria-Bertani broth and Tryptone Soy Broth, respectively, and shaken at 220 rpm for 16 h at 37°C. An aliquot of 100 µl overnight culture was inoculated into 10 ml fresh medium and shaken at 220 rpm, 37°C for 3.5 h until OD_600 nm_ reached 1.0. Bacteria cultures were collected by pelleting at 5000 g for 10 min, followed by washing with 145 mM saline, and the bacteria were resuspended to OD_600 nm_ = 10. Aliquots of 10 µl bacteria were collected by centrifugation. The *P. aeruginosa* was incubated with 10% patient or normal serum at 37°C for 60 min. The reaction was ceased on ice. The remnant bacterial population in the reaction mixture was enumerated by plating 100 µl of serially diluted samples on agar plates and incubating at 37°C overnight. To standardize the effect of CRP∶L-ficolin interaction in complement activation, and to exclude the effects of other mechanisms, a parallel experiment was set up with bacteria incubated in heat-inactivated serum, which served as a control to account for 0% killing. The % bacterial killing was calculated as follows:
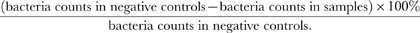



The real-time imaging of the bacterial clearance elicited by the serum (with or without CRP∶L-ficolin interaction) was conducted by mixing 10 µl aliquot of bacteria (*P. aeruginosa* PAO1-GFP or *S. aureus* PC1839-GFP) with 100 µl 10% serum. Three µl of the mixture was examined by real-time fluorescence microscopy (Axio observer Z1, Carl Zeiss Inc, Thornwood, NY). Images were taken at intervals of 15 s for 30 min, at magnification of 63×1.6 and a movie was made. Bacteria samples incubated with diluting buffers (TBS or MBS) served as the negative controls. To monitor the antibacterial effect over 30 min, the % bacteria killed (shown in the fluorescence image) was calculated as follows:
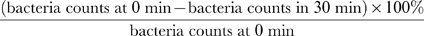



### Binding of proteins to *P. aeruginosa*

*P. aeruginosa* PAO1 (OD600 nm of 10 Units) was prepared as mentioned above. An aliquot of 100 µl of the bacteria was fixed with 5% (v/v) acetic acid for 5 min followed by washing thrice with TBS or MBS containing 0.05% v/v Tween-20 until the pH was stabilised to 7.4 or 6.5 in the respective buffers. The bacteria were then blocked with 1% (w/v) HSA in wash buffers (blocking buffers) under normal or infection-inflammation condition, for 2 h. To assess the binding of the purified proteins to the bacteria, 5 µg of purified CRP or 1.5 µg of purified L-/H- ficolins (diluted in 500 µl of blocking buffers under the two conditions) were incubated with the three-time washed bacteria at room temperature for 2 h. To study the effects of serum on the binding of CRP and/or L-/H- ficolins to the bacteria, after three washes, 5 µg of purified CRP or 1.5 µg of purified L-/H- ficolins (diluted in 500 µl of 10% (v/v) patient or healthy serum in the corresponding MBS or TBS buffers with 0.05% (v/v) Tween-20) were incubated with the bacteria at room temperature for 2 h. After 4 washes, proteins bound to the bacteria were eluted with 0.5 M ammonium formate (pH 6.4) and visualized by SDS PAGE and Western blot analysis.

### Co-immunoprecipitation (Co-IP)

Sera were diluted to 10% (v/v) in buffers containing 50 mM MES, pH 6.5 or 50 mM Tris, pH 7.4, 145 mM NaCl, 1% (v/v) NP-40 with 0, 2 or 2.5 mM CaCl_2_. Then 500 µl of the diluted sera were pre-cleared by incubating with 20 µl Protein A Sepharose (GE healthcare, Uppsala, Sweden) at 4°C for 1 h. Polyclonal antibody against human CRP was added to the supernatant and incubated for 1 h at 4°C with shaking. As a negative control, antibody against the horseshoe crab *Carcinoscorpius rotundicauda* CRP, CrCRP, which does not cross-react with human CRP [22], was added to a replicate supernatant. The inhibition effect of calcium on CRP∶L-ficolin interaction was analyzed by varying the calcium concentration at 0, 2 and 2.5 mM. Then, 20 µl protein A Sepharose was added to the supernatant and incubated for 1 h at 4°C with shaking. The beads were boiled in loading buffer and electrophoresed on 12% SDS-PAGE. The CRP∶L-ficolin complex on the beads was detected by Western blot.

### Real-time biointeraction analysis

BIAcore 2000 was used to demonstrate real-time biointeraction. PC-HPA chip was prepared by immobilizing 1-palmitoyl-2-oleoyl-phosphatidylcholine (Avanti Polar Lipids, Birmingham, AL) on HPA chip [63] (GE Healthcare). Then 200 nM CRP in running buffer was injected over the surface of PC-HPA followed by separate injections of 50, 100, and 200 µM L-rFBG under normal or infection-inflammation conditions at a flow rate of 30 µl/min. The running buffers for normal condition was 50 mM Tris, 145 mM NaCl and 2.5 mM calcium, pH 7.4, and for infection-inflammation condition, was 50 mM MES, 145 mM NaCl and 2 mM calcium, pH 6.5. The dissociation was for 180 s at the same flow rate. The PC-HPA chip was regenerated by injection of 15 µl of 0.1 M NaOH at 30 µl/min. Injection of binding buffer without CRP was used as the negative control. BIAevaluation 3.2 software was used to calculate the K_D_. All the surface plasmon resonance curves used in K_D_ calculation were normalized against negative controls.

### C4 cleavage assay

To achieve CRP-initiated complement activation, 20 µl immobilized PC beads (Pierce, Rockford, IL) was incubated at room temperature for 2 h with 500 µl protein solution in MBS, and TBS containing 10 µg/ml CRP, 3 µg/ml L-ficolin [64], 1 µg/ml MASP-2 [65], following which 1 µg/ml C4 [22] was added and incubated at room temperature for 1 h. For L-ficolin-initiated complement activation, a similar protocol was followed except that GlcNAc-Sepharose and 1 µg/ml C1 complex were used in place of PC-Sepharose and 1 µg/ml MASP-2. The negative controls were beads incubated with incomplete complement components. The beads were boiled in loading buffer and electrophoresed on 12% SDS PAGE. Polyclonal rabbit anti-C4c antibody (1∶1000) and goat anti-rabbit secondary antibody (1∶2000) was used to detect the deposited C4 by immunoblotting.

### Immobilization of the fluorosphere beads with PC and GlcNAc

Red or green fluorosphere beads of 1 µm diameter (Invitrogen) were conjugated with phosphorylcholine-BSA (PC-BSA) (Biosearch Technologies, Novato, CA) or GlcNAc-BSA (Dextra Laboratories, Reading, UK), respectively. The green or red beads conjugated with BSA alone served as reciprocal controls. After a brief sonication, an aliquot of 100 µl fluorosphere beads was incubated with 100 µl of 1 mg/ml PC-BSA, GlcNAc-BSA or BSA alone at room temperature for 15 min. Then, 0.8 mg of 1-ethyl-3-(3-dimethylaminopropyl)-carbodimide hydrochloride, EDC (GE Healthcare) was added to the reaction mixture, vortexed and incubated for 2 h at room temperature. Subsequently, glycine was added to a final concentration of 100 mM and incubated at room temperature for 30 min to stop the reaction. Beads were washed thrice with PBS and resuspended in 100 µl TBS or MBS.

### Phagocytosis assay

The PC- (red) or GlcNAc- (green) conjugated phagocytic fluorosphere beads were pretreated in the same way as for the PC and GlcNAc beads in the C4 cleavage assay to generate the opsonized particles. U937 cells were treated with 30 ng/ml phorbol myristate acetate for 24 h and the medium was refreshed. The cells were harvested 48 h later and collected by centrifugation, washed twice with PBS and resuspended in TBS and MBS buffers, and enumerated. Then, 1×10^5^ cells were incubated at 37°C for 15 min with 1.5×10^6^ opsonized red PC-beads together with an equal amount of green control beads. Similarly, 1×10^5^ cells were incubated at 37°C for 30 min with 1.5×10^6^ opsonized green GlcNAc-beads together with an equal amount of red control beads. Phagocytosis was stopped by adding 1 ml ice-cold PBS. Cells were collected and washed thrice with PBS and fixed in 4% paraformaldehyde in PBS. Three µl of the cell suspension was examined by fluorescence microscopy (BX 60, Olympus, Tokyo, Japan). For quantification of the phagocytic efficiency, three different fields were chosen. The number of cells and the number of phagocytosed beads were enumerated in each field. As the initial ratio of cells∶beads was 1∶15, the average of 15 beads in a cell was considered 100% phagocytosis. Thus the phagocytic efficiency was calculated as follows:
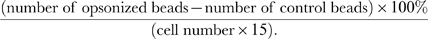



### ELISA to analyze direct protein–protein interaction

To test the interaction between L-rFBG and CRP, 0.8 µg of CRP in 100 µl coating buffer (50 mM sodium carbonate/bicarbonate buffer, pH 9.6) was immobilized on 96-well Maxisorp™ plates (NUNC, Roskilde, Denmark) by incubating overnight at 4°C. After three washes with TBST containing 25 mM Tris-HCl, pH 7.4, 145 mM NaCl, 0.05% (v/v) Tween-20, the wells were blocked with 1% (w/v) HSA in TBST (blocking buffer) at 37°C for 2 h. After four washes, 0.8 µg of L-rFBG (with myc fusion tag) in 100 µl blocking buffer was added to the wells and incubated at 37°C for 2 h. Following three washes, the bound L-rFBG was detected with anti-myc antibody (1∶1000) followed by rabbit anti-mouse HRP-conjugated secondary antibody (1∶2000). After adding ABTS substrate (Roche Diagnostics, Mannheim, Germany), the OD_405 nm_ was read. Other ELISA experiments were carried out similarly except for different immobilized proteins and different binding proteins which were detected with the corresponding primary and secondary antibodies. Wells coated with HSA instead of the immobilized proteins served as negative controls unless otherwise stated. It was reported that MBS was commonly used to adjust the acidic condition of the serum [43],[44]. Thus MBS containing 25 mM MES, 145 mM NaCl and 2 or 2.5 mM calcium adjusted to pH: 5.5, 6.0 and 6.5, and TBS containing 25 mM Tris-HCl, 145 mM NaCl and 2 or 2.5 mM calcium, adjusted to pH 7.0 and 7.4 were used as the binding buffers to examine the effect of pH on the protein-protein interaction.

For C1q and L-ficolin competition assay, 0.8 µg CRP in 100 µl coating buffer was immobilized on each well of the Maxisorp™ ELISA plate (NUNC) as mentioned above. After blocking for 2 h in 100 µl blocking buffer (TBS or MBS with 0.05% v/v Tween-20 and 1% w/v HSA), 0.8 µg of C1q with increasing amounts of L-ficolin, or 0.8 µg of L-ficolin with increasing amounts of C1q in 100 µl of blocking buffer was added to each well. Similarly, for MASP-2 and CRP competition assay, 0.8 µg of L-ficolin was immobilized, and 0.8 µg of MASP-2 with increasing amounts of CRP or 0.8 µg of CRP with increasing amounts of MASP-2 in the same binding buffer, were added to each well. The added proteins with constant amount were detected with anti-C1q (1∶1000), anti-L-ficolin (1∶1000), anti-MASP-2 (1∶1000) and anti-CRP (1∶1000) antibodies. Wells coated with HSA instead of CRP were the negative controls.

### Flow cytometry

The aliquoted *P. aeruginosa* PAO1 without GFP, was prepared similarly to that in the bacterial killing assay. Then the bacteria were fixed in 5% acetic acid for 5 min at room temperature, and washed with either TBS or MBS. The bacteria were blocked with 3% (w/v) HSA in TBS or MBS with 0.05% (v/v) Tween-20 (blocking buffer) at room temperature for 1 h with shaking. Following three washes, the bacteria were incubated at room temperature for 30 min with 10% (v/v) serum, 10% (v/v) ficolin-depleted serum, 10% (v/v) CRP-depleted serum and 10% (v/v) serum depleted of both CRP and ficolin in TBS or MBS buffer to represent normal or infection-inflammation conditions, respectively. After three washes with the corresponding binding buffer, the bacteria were collected and incubated with anti-C3d (1∶100) in blocking buffer for 30 min. After three washes, the bacteria were collected and incubated with R-Phycoerythrin conjugated goat anti-rabbit (1∶200) for 30 min on ice. The bacteria were washed 5 times and fixed with 4% paraformaldehyde for 15 min. After three washes, the bacteria were diluted in PBS (140 mM NaCl, 10 mM phosphate, 2.7 mM KCl, pH of 7.4) which was also the running buffer for the flow cytometry. The bacterial cells with bound C3 signal were collected by Cytomation Cyan LX (Dako A/S) and the counts were analyzed by WinMDI version 2.8. For quantification of the FACS data, the synergistic effect of CRP and ficolin, under infection-inflammation and normal conditions was assessed using (i) the whole serum, (ii) ficolin-depleted serum and (iii) CRP-depleted serum. The relative C3 deposition in each case was calculated by subtracting off the background reading of both CRP- and ficolin- depleted serum.

### Statistical analysis

Data represent means±s.e.m. of three independent experiments with triplicates each. *P* values of less than 0.05 and 0.01 were respectively considered significant and very significant by Student's *t* test.

## Supporting Information

Figure S1 The CRP level in the blood of patients diagnosed with infection compared to normal healthy individuals. (A) The CRP concentration in normal individuals and patients included in this study. (B) The average CRP levels from five healthy volunteers and nine patients were measured by Bioassay ELISA kit (BD Biosciences, San Jose, CA) and calculated to represent the CRP level in normal individuals (<0.05 µg/ml) and patients (10 µg/ml). The significant difference in CRP level confirmed inflammation in these patients. **: *P*<0.01 by Student's t test.
(0.26 MB TIF)

Figure S2 In vitro bacteria killing assay. (A) Bright field of bacterial killing effect image (Figure 1A) indicated that the bacteria were within the field of view. 10^8^ cfu bacteria with GFP fluorescence were incubated with sera or buffers under normal or infection-inflammation conditions. Images were taken at 15 s intervals for 30 min (magnification: 63×1.6). Scale bar represents 20 µm. (B) The endpoint bactericidal activity of the TBS (pH = 7.4, 2.5 mM calcium) and MBS (pH = 6.5, 2 mM calcium) buffers were analyzed by incubating 10^8^ cfu bacteria with TBS or MBS for 1 h. Same amount of bacteria were then plated on the LB plates and incubated at 37°C for 16 h. The remnant bacteria were enumerated and the bacterial killing rate in the two buffers was calculated as the percentage of the 100% survival.
(7.32 MB TIF)

Figure S3 The alignment of three ficolin isoforms using Bioedit Sequence Alignment Editor. L- and M- ficolin sequences show high homology with each other while both of them show low homology to H-ficolin.
(0.08 MB TIF)

Figure S4 The purified ficolins. Lane 1: protein marker. Lane 2: recombinant L-rFBG (27 kDa). Lane 3: recombinant M-ficolin (37 kDa). Lanes 5–6: native L-ficolin (35 kDa) and H-ficolin (37 kDa) purified from human plasma. The purified proteins were resolved on 12% SDS PAGE and stained with Commassie-blue.
(0.43 MB TIF)

Figure S5 Confirmation of the interaction between CRP and ficolins. (A) ELISA shows that CRP does not interact with H-ficolin independent of calcium. Two different orders of immobilization were tested: either CRP was immobilized first or H-ficolin (HFL) was immobilized first. In both positions, H-ficolin was not able to bind to CRP. (B) Yeast-two hybrid assay to check whether CRP interacts with M-ficolin. Colonies growing on the SD-Trp-Leu plate indicate the successful transformation. Growth on the QDO plates indicates interaction between CRP and M-ficolin. Transformation of the empty vector plasmid with the protein of interest excludes the possibility of autoactivation. pY1GAL4 plasmid was used as a positive control.
(1.23 MB TIF)

Figure S6 Phagocytosis of the opsonized beads. In normal condition, even when all the amplification pathway components were added, they were not functional due to lack of interaction between CRP and L-ficolin. In infection-inflammation condition, addition of all the amplification pathway components showed the fluorescence signal due to CRP∶L-ficolin interaction, whereas all the controls do not display any fluorescence signal. The image was taken with an Olympus Fluorescence Microscope (BX 60; magnification: 400×).
(6.45 MB TIF)

Figure S7 Purity of human C-reactive protein. (A) One µg of CRP was resolved on 12% reducing SDS PAGE and stained with Commassie-blue, showing ∼98% purity. (B) Mass spectrometry of the CRP. One µg of CRP was trypsin-digested and analyzed by MALDI-TOF-TOF to check for purity. Within the significant range (*P*<0.05), CRP was substantially pure. Grey boxes show the fragments that are consistent with CRP fingerprints in the database.
(0.83 MB TIF)

Protocol S1 Supporting Materials and Methods
(0.05 MB DOC)

Video S1 The real-time imaging of bacterial clearance elicited by serum components in normal and infection-inflammation conditions–Video S1: PAO1-pDSK incubated with 10% patient serum in infection-inflammation condition.
(3.18 MB MOV)

Video S2 The real-time imaging of bacterial clearance elicited by serum components in normal and infection-inflammation conditions–Video S2: PAO1-pDSK incubated with 10% healthy serum in normal condition.
(2.26 MB MOV)

Video S3 The real-time imaging of bacterial clearance elicited by serum components in normal and infection-inflammation conditions–Video S3: PAO1-pDSK incubated with MBS buffer only.
(3.21 MB MOV)

Video S4 The real-time imaging of bacterial clearance elicited by serum components in normal and infection-inflammation conditions–Video S4: PAO1-pDSK incubated with TBS buffer.
(2.00 MB MOV)

Video S5 The real-time imaging of bacterial clearance elicited by serum components in normal and infection-inflammation conditions–Video S5: PAO1-pDSK incubated with 10% heat inactivated patient serum in infection-inflammation condition.
(2.17 MB MOV)

Video S6 The real-time imaging of bacterial clearance elicited by serum components in normal and infection-inflammation conditions–Video S6: PC1389-pALC incubated with 10% patient serum in infection-inflammation condition.
(5.58 MB MOV)
