# Supplementary figures and images for: Local Inflammation Induces Complement Crosstalk Which Amplifies the Antimicrobial Response
Source: PLoS Pathog. 2009 Jan 30;5(1):e1000282. doi: 10.1371/journal.ppat.1000282 (PMC2629585; doi:10.1371/journal.ppat.1000282)

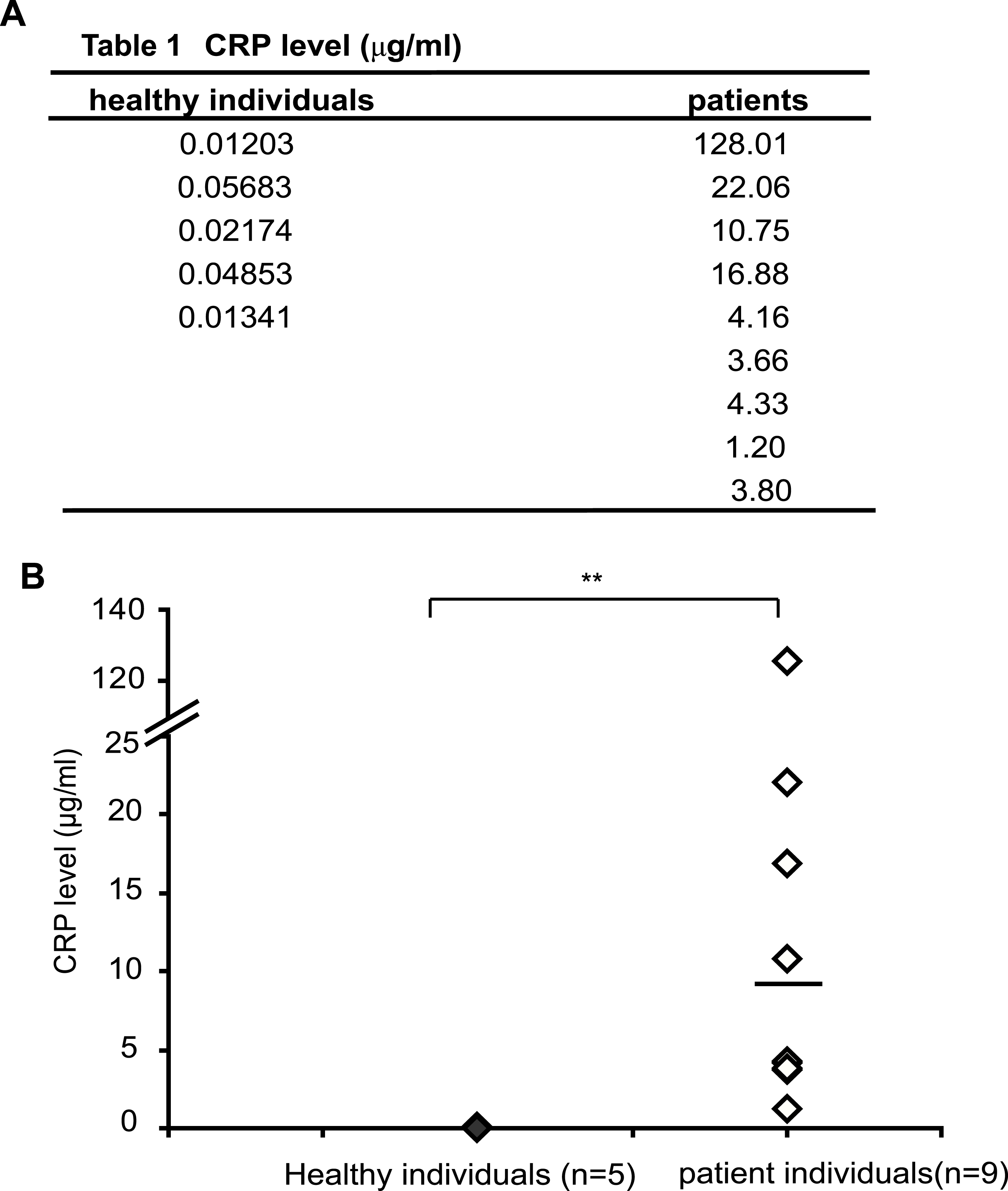

Supplement: Figure S1 — The CRP level in the blood of patients diagnosed with infection compared to normal healthy individuals. (A) The CRP concentration in normal individuals and patients included in this study. (B) The average CRP levels from five healthy volunteers and nine patients were measured by Bioassay ELISA kit (BD Biosciences, San Jose, CA) and calculated to represent the CRP level in normal individuals (<0.05 µg/ml) and patients (10 µg/ml). The significant difference in CRP level confirmed inflammation in these patients. **: P<0.01 by Student's t test. (0.26 MB TIF) [file ppat.1000282.s001.tif]

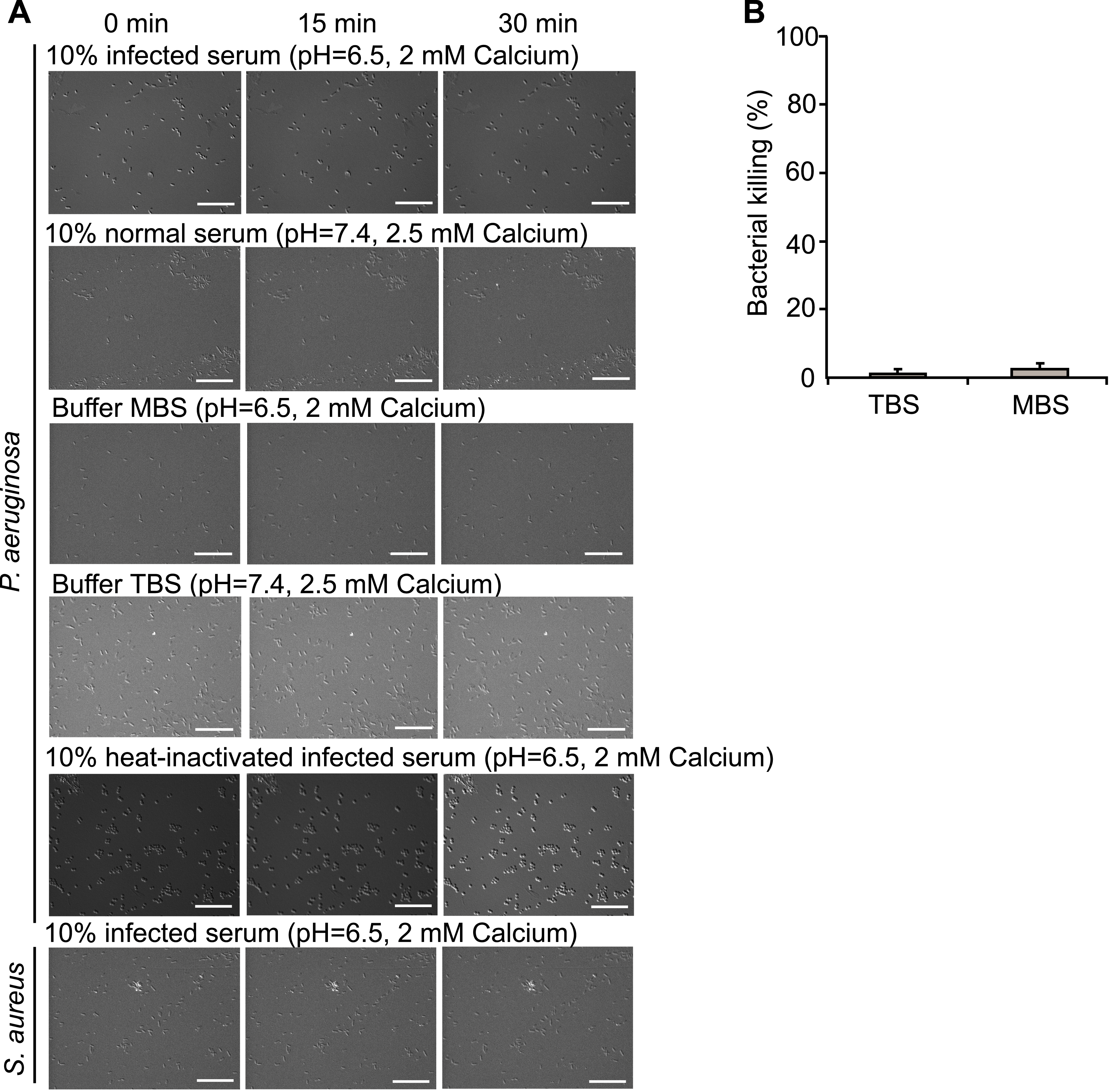

Supplement: Figure S2 — In vitro bacteria killing assay. (A) Bright field of bacterial killing effect image (Figure 1A) indicated that the bacteria were within the field of view. 108 cfu bacteria with GFP fluorescence were incubated with sera or buffers under normal or infection-inflammation conditions. Images were taken at 15 s intervals for 30 min (magnification: 63×1.6). Scale bar represents 20 µm. (B) The endpoint bactericidal activity of the TBS (pH = 7.4, 2.5 mM calcium) and MBS (pH = 6.5, 2 mM calcium) buffers were analyzed by incubating 108 cfu bacteria with TBS or MBS for 1 h. Same amount of bacteria were then plated on the LB plates and incubated at 37°C for 16 h. The remnant bacteria were enumerated and the bacterial killing rate in the two buffers was calculated as the percentage of the 100% survival. (7.32 MB TIF) [file ppat.1000282.s002.tif]

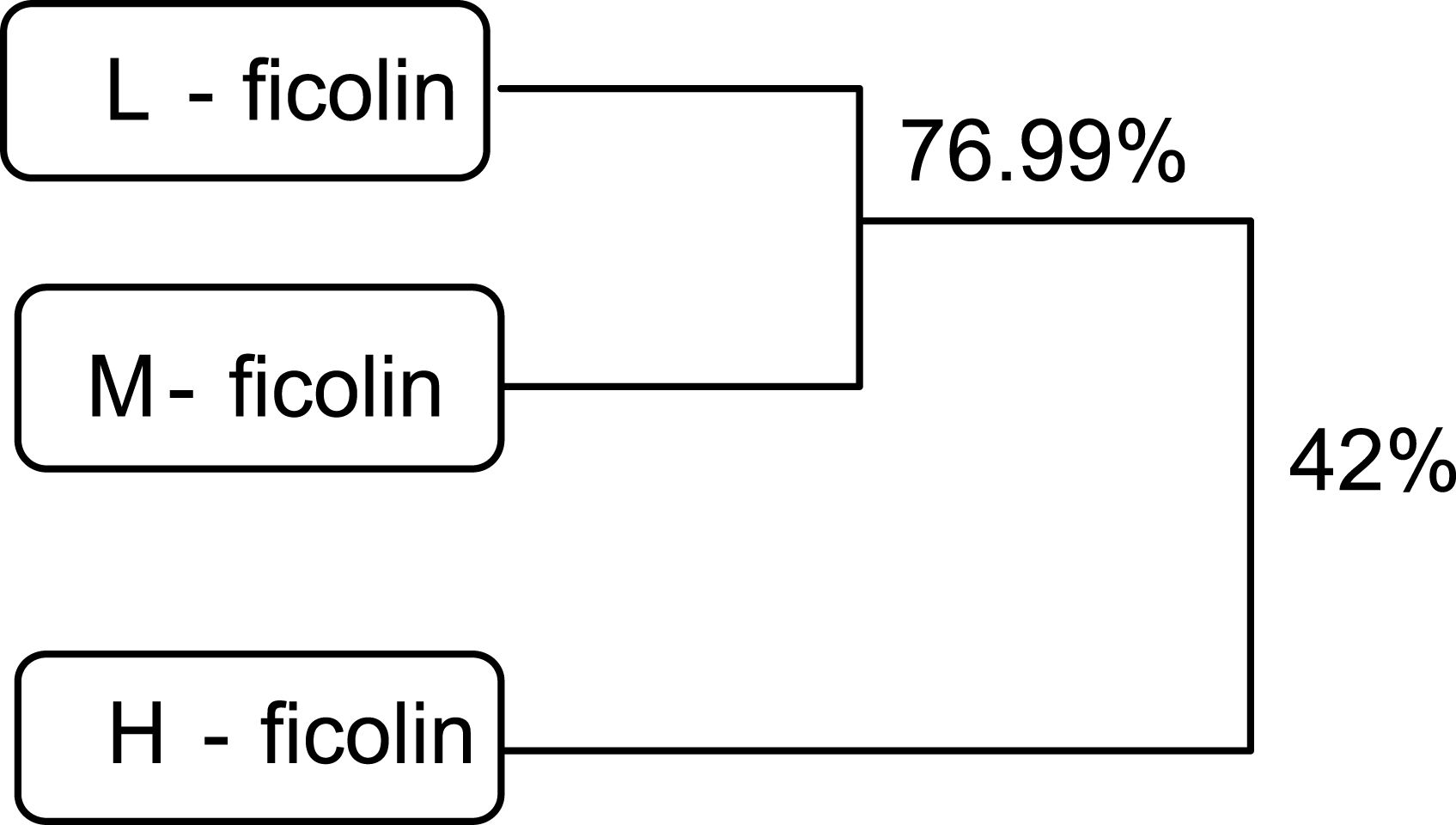

Supplement: Figure S3 — The alignment of three ficolin isoforms using Bioedit Sequence Alignment Editor. L- and M- ficolin sequences show high homology with each other while both of them show low homology to H-ficolin. (0.08 MB TIF) [file ppat.1000282.s003.tif]

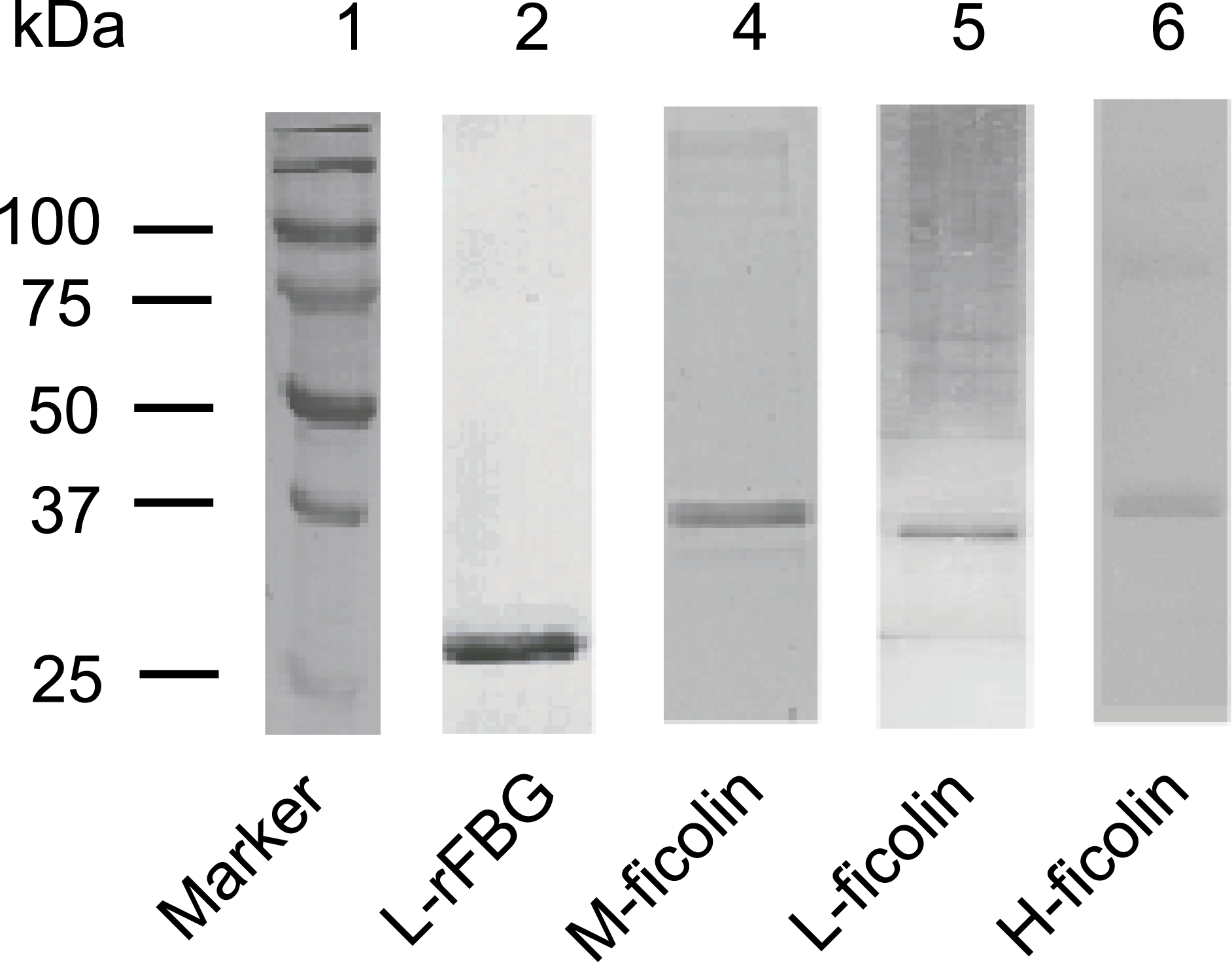

Supplement: Figure S4 — The purified ficolins. Lane 1: protein marker. Lane 2: recombinant L-rFBG (27 kDa). Lane 3: recombinant M-ficolin (37 kDa). Lanes 5–6: native L-ficolin (35 kDa) and H-ficolin (37 kDa) purified from human plasma. The purified proteins were resolved on 12% SDS PAGE and stained with Commassie-blue. (0.43 MB TIF) [file ppat.1000282.s004.tif]

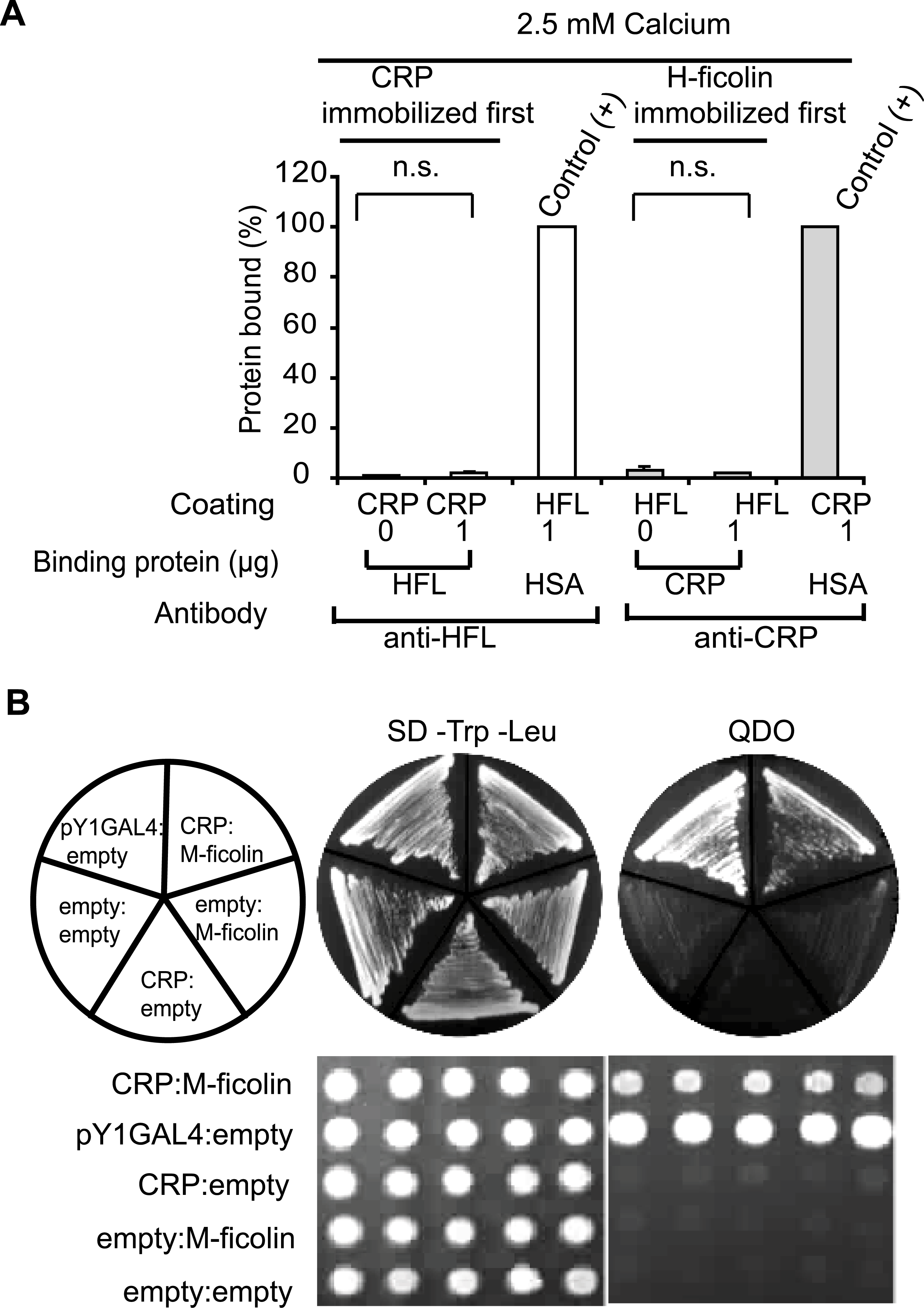

Supplement: Figure S5 — Confirmation of the interaction between CRP and ficolins. (A) ELISA shows that CRP does not interact with H-ficolin independent of calcium. Two different orders of immobilization were tested: either CRP was immobilized first or H-ficolin (HFL) was immobilized first. In both positions, H-ficolin was not able to bind to CRP. (B) Yeast-two hybrid assay to check whether CRP interacts with M-ficolin. Colonies growing on the SD-Trp-Leu plate indicate the successful transformation. Growth on the QDO plates indicates interaction between CRP and M-ficolin. Transformation of the empty vector plasmid with the protein of interest excludes the possibility of autoactivation. pY1GAL4 plasmid was used as a positive control. (1.23 MB TIF) [file ppat.1000282.s005.tif]

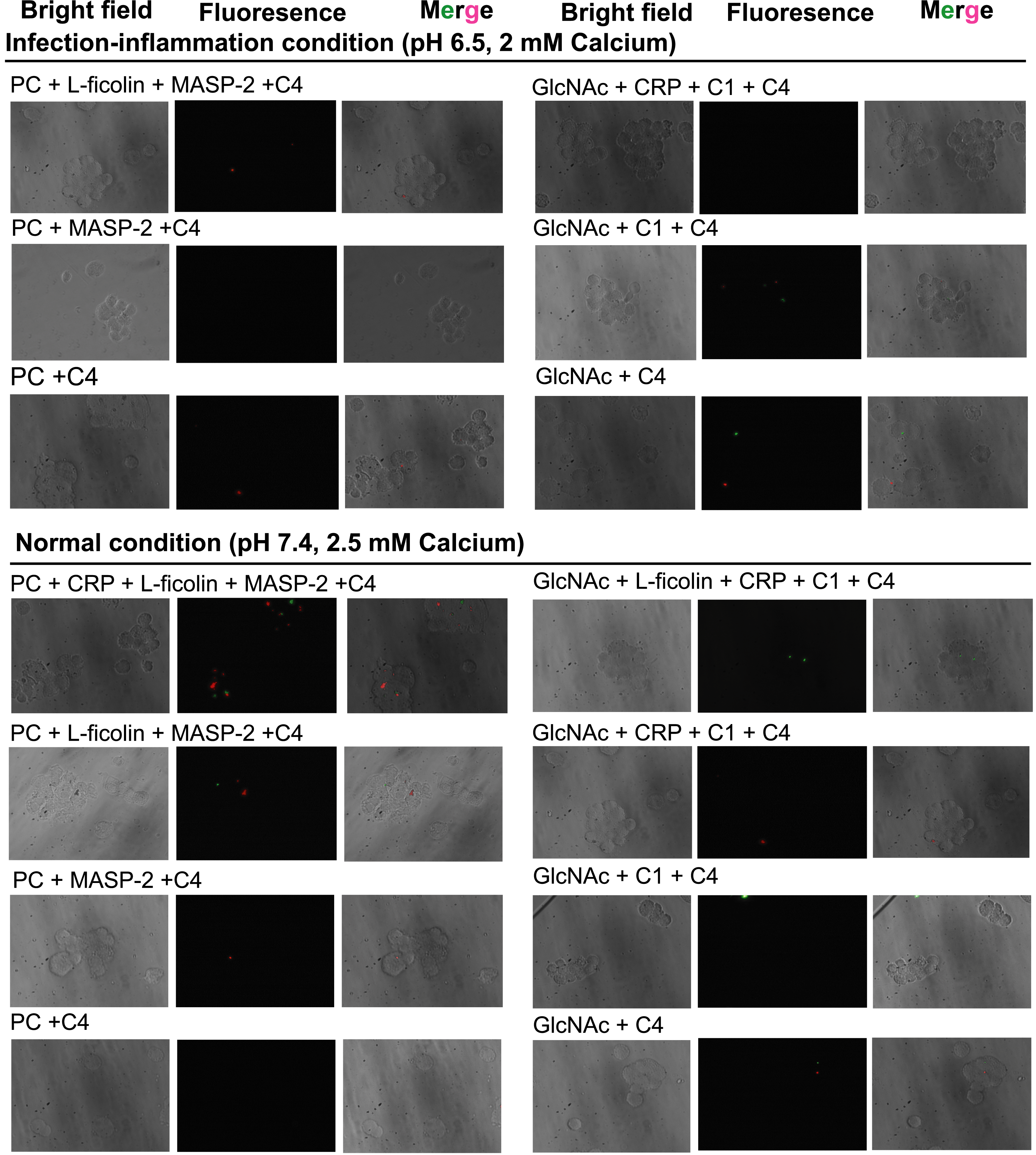

Supplement: Figure S6 — Phagocytosis of the opsonized beads. In normal condition, even when all the amplification pathway components were added, they were not functional due to lack of interaction between CRP and L-ficolin. In infection-inflammation condition, addition of all the amplification pathway components showed the fluorescence signal due to CRP∶L-ficolin interaction, whereas all the controls do not display any fluorescence signal. The image was taken with an Olympus Fluorescence Microscope (BX 60; magnification: 400×). (6.45 MB TIF) [file ppat.1000282.s006.tif]

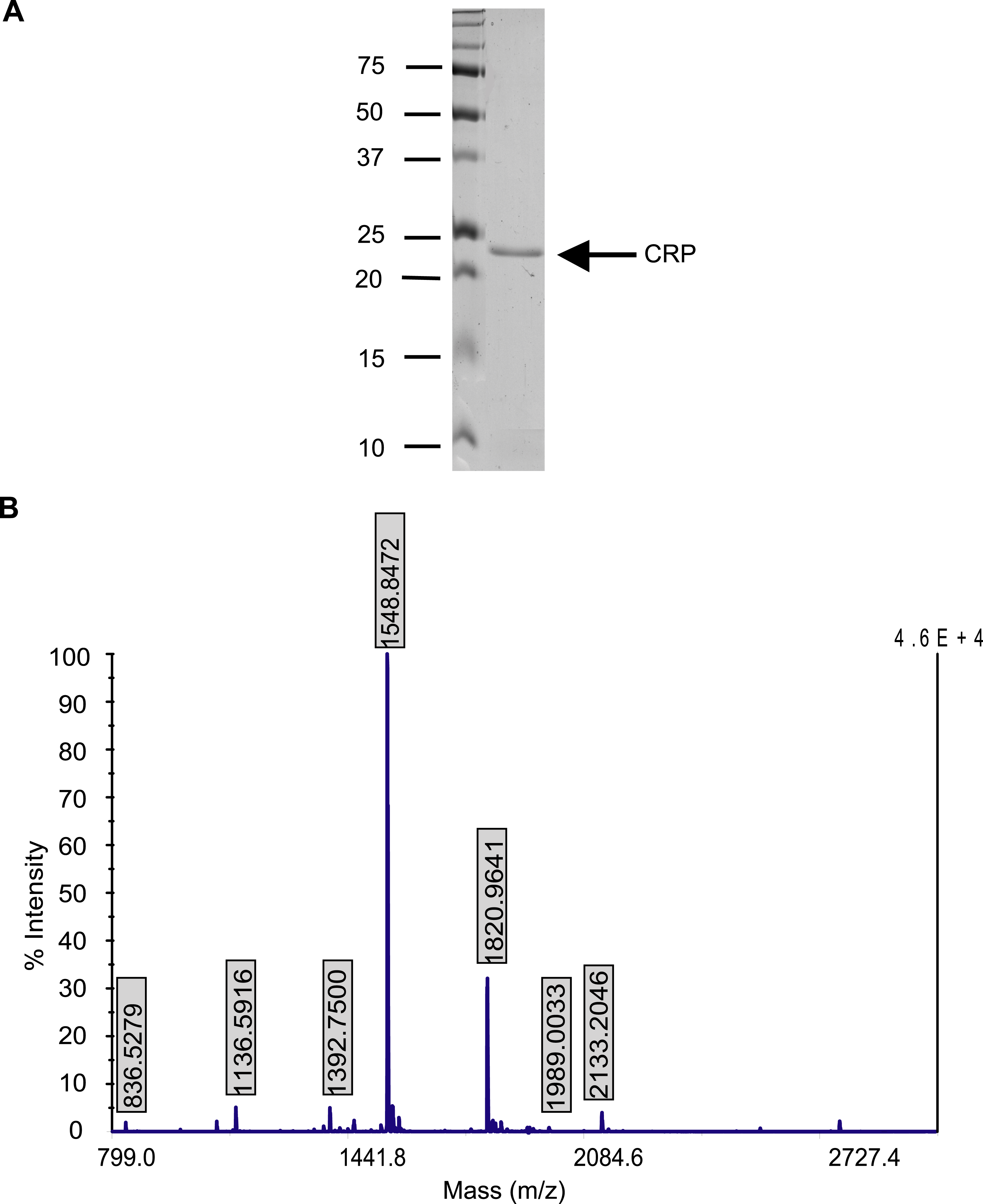

Supplement: Figure S7 — Purity of human C-reactive protein. (A) One µg of CRP was resolved on 12% reducing SDS PAGE and stained with Commassie-blue, showing ∼98% purity. (B) Mass spectrometry of the CRP. One µg of CRP was trypsin-digested and analyzed by MALDI-TOF-TOF to check for purity. Within the significant range (P<0.05), CRP was substantially pure. Grey boxes show the fragments that are consistent with CRP fingerprints in the database. (0.83 MB TIF) [file ppat.1000282.s007.tif]
