# Supplementary material for: Local Inflammation Induces Complement Crosstalk Which Amplifies the Antimicrobial Response
Source: PLoS Pathog. 2009 Jan 30;5(1):e1000282. doi: 10.1371/journal.ppat.1000282 (PMC2629585; doi:10.1371/journal.ppat.1000282)
Supplement: Protocol S1 — Supporting Materials and Methods (0.05 MB DOC) [file ppat.1000282.s008.doc]

# SUPPLEMENTARY MATERIALS AND METHODS

**Mass spectrometry**

Protein bands were excised from the SDS PAGE and in-gel digested with trypsin [1]. Peptide mass fingerprint was obtained by MALDI-TOF-TOF (Voyager-DE^TM^ STR Biospectrometry^TM^ Workstation, Perseptive Biosystems, Framingham, MA). Peptide fragments were sequenced from the website of Matrix Science.

**Measurement of CRP concentration in human serum**

CRP concentrations in human serum were measured by human CRP BioAssay^TM^ ELISA kit (BD Biosciences) based on the manufacturer’s recommended protocol. Briefly, the samples were diluted 10-50 times before the measurement and the standards were added to the 96-well plate pre-coated with anti-CRP. After 30 min incubation at 37 ^o^C, the wells were washed five times using the wash buffer. Then, 100 µl HRP-conjugated anti-CRP antibody was added and incubated for 30 min. After 5 washes, 100 µl of substrate was added followed by incubation for 15 min at 37 ^o^C. The reaction was stopped with 100 µl stop solution, and the OD_450nm_ was read using SpectraMax 340 microtiter plate reader (Molecular devices, Sunnyvale, CA).

**Expression and Purification of L-rFBG.**

Functional fragment of the FBG domain of L-ficolin was cloned into the pSectag 2C vector (Invitrogen). The clone was transfected into HEK 293 cells by lipofectamine 2000 (Invitrogen) according to the product instruction. The medium was collected at 48 h after transfection. 300 μl Ni-NTA beads (Qiagen, Valencia, CA) were added to 30 ml medium and incubated at 4 ^o^C overnight. Then the beads were packed into the 10 ml poly-prep^TM^ chromatography column (Biorad, Hercules, CA). 50 ml washing buffer consisting of 20 mM Tris, 500 mM NaCl, pH = 8.0 with 20 mM imidazole (Sigma) was passed through the column. Using elution buffer (20 mM Tris, 500 mM NaCl and 250 mM imidazole, pH=8.0), the protein was eluted in 6 fractions of 120 μl each. The purity of the recombinant protein was tested by SDS PAGE.

**Purification of M-ficolin**

The full length M-ficolin cDNA was cloned into the mammalian expression vector pCDNA3.1 without His/V5 (Invitrogen). The clone was transfected into HEK 293 cells by Lipofectamine 2000 (Invitrogen). The medium was collected at 48 h after transfection and passed through GlcNAc Sepharose (Sigma). Protein was eluted with 200 mM GlcNAc and ultrafiltered through Vivaspin column (Sartorius Stedim Biotech, Aubagne Cedex, France) to remove GlcNAc, and the buffer was exchanged into 25 mM Tris-HCl, pH 7.4, 145 mM NaCl. The purity of the recombinant protein was tested by SDS PAGE (**Figure S4,** lane 3).

**Purification of native L-ficolin**

L-ficolin was purified [2] by concentrating normal human serum by a 4-8% PEG 6000 cut. After centrifugation, the pellet was dissolved in 0.5 M NaCl, 1.5 mM NaN_3_, 2 mM EDTA, 0.01% (v/v) polyoxyethylene 10-tridecyl ether (Sigma) and loaded at a flow rate of 0.5 ml/min onto an 8-ml column packed with Sepharose 4B (GE healthcare) coupled with *N*-acetylcysteine (Sigma) [2]. After washing, the bound L-ficolin was eluted with 10 mM Tris-HCl, pH 7.4, 20 mM NaCl, 1.5 mM NaN_3_, 2 mM EDTA, 0.01% (v/v) Emulfogen. The fractions with the highest protein recovery were pooled and passed through a 1 ml MonoQ column. The bound protein was eluted by a gradient of 50 - 500 mM NaCl in 20 ml volume. SDS PAGE was used to check the purity of L-ficolin (**Figure S4**, lane 4).

**Purification of native H-ficolin**

The precipitated healthy serum proteins from 4-8% PEG 6000 cut was dissolved in 10 mM Tris-HCl, pH 7.4 containing 145 mM NaCl, 10 mM CaCl_2_ and 0.05% (v/v) Tween-20 (running buffer), and chromatographed through an 8-ml human serum albumin (HSA)-conjugated Sepharose 4B column [3]. Then the eluate was passed through an 8-ml column containing acetylated-HSA-Sepharose 4B beads [3]. The column was sequentially washed with running buffer followed by 200 mM GlcNAc in running buffer. The bound H-ficolin was eluted in two steps: with 1 M Na-acetate, 0.05% Tween 20, 10 mM CaCl_2_, pH 7.5 followed by 10 mM diethylamine, 0.05% Tween 20, 10 mM CaCl_2_, pH 11.5. The latter eluate was neutralized with 10 mM Tris-HCl, pH 7.4. The eluted proteins were concentrated through a 1 ml MonoQ column (GE healthcare). The bound proteins were eluted with a second buffer containing 1 M NaCl. The eluate was chromatographed through a HighLoad Superdex G200 column (GE healthcare) to exchange the buffer to 10 mM Tris-HCl, pH 7.4, 145 mM NaCl. SDS PAGE confirmed the protein purity (**Figure S4**, lane 5).

**SDS-PAGE and Western blot**

Protein samples were boiled at 95 ^o^C for 5 min and electrophoresed on 12% SDS-PAGE and transferred to PVDF (BioRad) membrane for 1 h at 70V. The membrane was blocked with 3% (w/v) skimmed milk in 50 mM Tris, pH 7.4, 145 mM NaCl, 0.05% (v/v) Tween-20 (TBST) for 2 h. The membrane was probed with anti-hCRP (1:1000), anti-L-ficolin (1:1000), anti-c-myc (1:5000), anti-C4c (1:1000), anti-C3d (1:1000), anti-H-ficolin (1:1000) and anti-M-ficolin (1:1000). After four repeated washes with TBST, the membrane was probed with the corresponding secondary antibody in the recommended titer for 1 h, and washed four times in TBST. The immunosignals were detected using Supersignal West Pico Chemiluminescent Substrate (Pierce) and exposed to X-ray film (Fujifilm, Tokyo, Japan).

**References:**

1. Ng PM, Jin Z, Tan SS, Ho B, Ding JL (2004) C-reactive protein: a predominant LPS-binding acute phase protein responsive to Pseudomonas infection. J Endotoxin Res 10: 163-174.

2. Krarup A, Thiel S, Hansen A, Fujita T, Jensenius JC (2004) L-ficolin is a pattern recognition molecule specific for acetyl groups. J Biol Chem 279: 47513-47519.

3. Tachikawa T, Yazawa S, Asao T, Shin S, Yanaihara N (1991) Novel method for quantifying alpha(1----3)-L-fucosyltransferase activity in serum. Clin Chem 37: 2081-2086.
